# Supplementary material for: Barriers and enablers for implementation of clinical practice guidelines in maternity and neonatal settings: A rapid review
Source: PLoS One. 2024 Dec 16;19(12):e0315588. doi: 10.1371/journal.pone.0315588 (PMC11649122; doi:10.1371/journal.pone.0315588)
Supplement: S1 File — (DOCX) [file pone.0315588.s001.docx]

**Supplementary File S1 Full text screening summary**

|  | Full-text screening and study selection |  |  |  |
| --- | --- | --- | --- | --- |
|  | Authors | Published Year | Title | Include/Exclude |
| 1 | Pangerl S; Sundin D; Geraghty S | 2021 | Group B Streptococcus Screening Guidelines in Pregnancy: A Critical Review of Compliance. | Include |
| 2 | Gkentzi D; Katsakiori P; Marangos M; Hsia Y; Amirthalingam G; Heath PT; Ladhani S | 2017 | Maternal vaccination against p O'Loughlin F.; Phangmanixay S.; Sisouk K.; Phommanivong V.; Phiahouaphanh O.; Al Eryani S.; Raajimakers H.; Gray A.  Trevisanuto, Daniele; Marchetto, Luca; Arnolda, Gaston; Chien, Tran Dinh; Lincetto, Ornella; Cavallin, Francesco; Xuan, Ngo Minh; Tien, Nguyen Viet; Hoi, Nguyen Thi Xuan; Moccia, Luciano  ertussis: a systematic review of the recent literature. | Include |
| 3 | Nair M; Yoshida S; Lambrechts T; Boschi-Pinto C; Bose K; Mason EM; Mathai M | 2014 | Facilitators and barriers to quality of care in maternal, newborn and child health: a global situational analysis through metareview. | Include |
| 4 | Ryan N; Vieira D; Goffman D; Bloch EM; Akaba GO; D'mello BS; Egekeze C; Snyder A; Lyimo M; Nnodu O; Peprah E | 2020 | Implementation outcomes of policy and programme innovations to prevent obstetric haemorrhage in low- and middle-income countries: a systematic review. | Include |
| 5 | Doherty E; Kingsland M; Elliott EJ; Tully B; Wolfenden L; Dunlop A; Symonds I; Attia J; Ward S; Hunter M; Azzopardi C; Rissel C; Gillham K; Tsang TW; Reeves P; Wiggers J | 2022 | Practice change intervention to improve antenatal care addressing alcohol consumption during pregnancy: a randomised stepped-wedge controlled trial. | Include |
| 6 | Kebaya LMN; Kiruja J; Maina M; Kimani S; Kerubo C; McArthur A; Munn Z; Ayieko P | 2018 | Basic newborn resuscitation guidelines for healthcare providers in Maragua District Hospital: a best practice implementation project. | Include |
| 7 | Nkamba D; Mwenechanya M; Kilonga AM; Cafferata ML; Berrueta AM; Mazzoni A; Althabe F; Garcia-Elorrio E; Tshefu AK; Chomba E; Buekens PM; Belizan M | 2017 | Barriers and facilitators to the implementation of antenatal syphilis screening and treatment for the prevention of congenital syphilis in the Democratic Republic of Congo and Zambia: results of qualitative formative research. | Include |
| 8 | Haskell L; Tavender EJ; O'Brien S; Wilson CL; Babl FE; Borland ML; Schembri R; Orsini F; Cotterell E; Sheridan N; Oakley E; Dalziel SR | 2021 | Process evaluation of a cluster randomised controlled trial to improve bronchiolitis management - a PREDICT mixed-methods study. | Include |
| 9 | Turan JM; Steinfeld RL; Onono M; Bukusi EA; Woods M; Shade SB; Washington S; Marima R; Penner J; Ackers ML; Mbori-Ngacha D; Cohen CR | 2012 | The study of HIV and antenatal care integration in pregnancy in Kenya: design, methods, and baseline results of a cluster-randomized controlled trial. | Include |
| 10 | Snelgrove-Clarke E; Davies B; Flowerdew G; Young D | 2015 | Implementing a Fetal Health Surveillance Guideline in Clinical Practice: A Pragmatic Randomized Controlled Trial of Action Learning. | Include |
| 11 | Zahroh RI; Hazfiarini A; Eddy KE; Vogel JP; TunÃ§alp Ó¦; Minckas N; Althabe F; Oladapo OT; Bohren MA | 2022 | Factors influencing appropriate use of interventions for management of women experiencing preterm birth: A mixed-methods systematic review and narrative synthesis. | Include |
| 12 | Luitjes SHE; Hermens RPMG; de Wit L; Heymans MW; van Tulder MW; Wouters MGAJ. | 2018 | An innovative implementation strategy to improve the use of Dutch guidelines on hypertensive disorders in pregnancy: A randomized controlled trial. | Include |
| 13 | Gu Y.; HY; Zhang H.; FW; Yang Y; Latour JM. | 2020 | Implementation of an Evidence-Based Guideline of Enteral Nutrition for Infants With Congenital Heart Disease: A Controlled Before-and-After Study | Include |
| 14 | da Silva Carvalho V.K; da Silva EN; Barreto JOM. | 2021 | Public engagement in health technology assessment in Brazil: the case of the public consultation on National Clinical Guidelines for Care in Normal Birth | Include |
| 15 | Sharma,A; Birkeland K.I; Nermoen I; Qvigstad E; Tran AT; Gulseth HL; Sollid ST; Wium C; Varsi C. | 2021 | Understanding mechanisms behind unwanted health behaviours in Nordic and South Asian women and how they affect their gestational diabetes follow-ups: A qualitative study | Include |
| 16 | Rousseau, A; Azria, E; Baumann, S; Deneux-Tharaux, C; Senat, MV. | 2020 | Do obstetricians apply the national guidelines? A vignette-based study assessing practices for the prevention of preterm birth | Include |
| 17 | Muirhead, R.; Kynoch, K. | 2019 | Implementation of an opioid weaning protocol to improve pain management, and to prevent or decrease iatrogenic withdrawal syndrome in the neonatal intensive care | Include |
| 18 | Muhumuza, C; Gomersall, JS; Fredrick, ME; Atuyambe, L; Okiira, C; Mukose, A; Ssempebwa, J. | 2015 | Health care worker hand hygiene in the pediatric special care unit at Mulago National Referral Hospital in Uganda: a best practice implementation project | Include |
| 19 | Warren, D. | 2011 | Implementation of a protocol for the prevention and management of extravasation injuries in the neonatal intensive care patient | Include |
| 20 | Alsweiler, JM; Gomes, L; Nagy, T; Gilchrist, CA; Hegarty, JE. | 2020 | Adherence to neonatal hypoglycaemia guidelines: A retrospective cohort study. | Include |
| 21 | Trollope, H; Leung, JPY; Wise, M; Farquhar, C; Sadler, L. | 2018 | An evaluation of the objective quality and perceived usefulness of maternity clinical practice guidelines at a tertiary maternity unit. | Include |
| 22 | de Oliveira C, Amanda C; Saraiva, ARB; GonÃ§alves, GA A; Soares, JR; de Lima Pinto, S. | 2013 | Breastfeeding: Providing care in rooming-in care. | Include |
| 23 | Sundercombe, SL; Raynes-Greenow, CH; Turner, RM; Jeffery, HE. | 2014 | Do neonatal hypoglycaemia guidelines in Australia and New Zealand facilitate breast feeding? | Include |
| 24 | Mohan, DR; Lu, H; McClary, J; Marasch, J; Nock, ML; Ryan, RM. | 2023 | Evaluation of Intravenous Immunoglobulin Administration for Hyperbilirubinemia in Newborn Infants with Hemolytic Disease. | Include |
| 25 | SkÃ¥re, C; Calisch, TE; SÃ¦ter, E; Rajka, T; Boldingh, A.; Nakstad, B; Niles, DE; Kramerâ Johansen, J; Olasveengen, TM. Saeter, E; Kramer-Johansen, J. | 2018 | Implementation and effectiveness of a video-based debriefing programme for neonatal resuscitation. | Include |
| 26 | Telfer, M; Illuzzi, J.; Jolles, D. | 2021 | Implementing an Evidence-Based Bundle to Reduce Early Labor Admissions and Increase Adherence to Labor Arrest Guidelines: A Quality Improvement Initiative. | Include |
| 27 | Olsen, SL; Park, ND; Tracy, K; Younger, D; Anderson, B. | 2018 | Implementing Standardized Feeding Guidelines, Challenges, and Results. | Include |
| 28 | Pauws, S; Zanten, H.; Beks, E; Lopriore, E; Pas, A; Stenson, B; van Zanten, HA; Pauws, SC; Beks, EC; Stenson, BJ; Te Pas, AB. | 2017 | Improving manual oxygen titration in preterm infants by training and guideline implementation. | Include |
| 29 | Lyngstad, LT; Steinnes, S; Le Marechal, F. | 2022 | Improving pain management in a neonatal intensive care unit with single family room. A quality improvement project. | Include |
| 30 | Albouy-Llaty, M; Nadeau, C; Descombes, E; Pierre, F.; Migeot, V. | 2012 | Improving perinatal Group B streptococcus screening with process indicators. | Include |
| 31 | David, R.; Evans, R.; Fraser, HSF. | 2021 | Modelling Prenatal Care Pathways at a Central Hospital in Zimbabwe. | Include |
| 32 | Page, D; Gilroy, M; Hurrion, E; Clark, L; Wilkinson, S. | 2017 | Optimising early neonatal nutrition using translational research methodology. | Include |
| 33 | Akuma, A.O; Jordan, S. | 2012 | Pain management in neonates: a survey of nurses and doctors. | Include |
| 34 | Langley, JM; Pelude, L; Durand, J; Embree, J; Forgie, S; Ivany, A; Le Saux, N; Matlow, A; Mertz, D; Moore, D; Mulvey, MR; Mounchili, A; Oleksen, K; Thomas, E; Vayalumkal, J; Zhang, J. | 2015 | Preventing Methicillin-Resistant Staphylococcus aureus (MRSA) transmission in pediatric health care facilities - a Canadian Nosocomial Infection Surveillance Program (CNISP) cross-sectional survey. | Include |
| 35 | Laubscher, B; BÃ¤nziger, O; Schubiger, G. | 2013 | Prevention of vitamin K deficiency bleeding with three oral mixed micellar phylloquinone doses: results of a 6-year (2005-2011) surveillance in Switzerland. | Include |
| 36 | Pricilla, RA; Brown, M; Wexler, C; Maloba, M; Gautney, Brad J; Finocchario-Kessler, S. | 2018 | Progress Toward Eliminating Mother to Child Transmission of HIV in Kenya: Review of Treatment Guidelines Uptake and Pediatric Transmission Between 2013 and 2016â€”A Follow Up. | Include |
| 37 | Moore, G; Reszel, J; Daboval, T; Lemyre, B; Barker, C; Dunn, S. | 2020 | Qualitative evaluation of a guideline supporting shared decision making for extreme preterm birth. | Include |
| 38 | Breakell, R; Thorndyke, B.; Clennett, J.; Harkensee, C. | 2018 | Reducing unnecessary chest X-rays, antibiotics and bronchodilators through implementation of the NICE bronchiolitis guideline. | Include |
| 39 | Alja'freh, SM; Abu-Shaikha, L. | 2021 | Self-Reported Adherence to the Use of Clinical Practice Guidelines of Hypertensive Disorders of Pregnancy in Jordanian Hospitals. | Include |
| 40 | Wilkinson, SA; Donaldson, E; Beckmann, M; Stapleton, H. | 2017 | Service-wide management of healthy gestational weight gain following an implementation science approach. | Include |
| 41 | Brozanski, BS; Piazza, AJ; Chuo, J; Natarajan, G; Grover, Theresa R; Smith, JR; Mingrone, T. | 2020 | STEPP IN: Working Together to Keep Infants Warm in the Perioperative Period. | Include |
| 42 | Brower, LH; Wilson, PM. Murtagh K, Eileen; Haslam, D; Courter, J; Goyal, N; Durling, M; Shah, SS; Schondelmeyer, A. | 2019 | Using Quality Improvement to Implement a Standardized Approach to Neonatal Herpes Simplex Virus. | Include |
| 43 | Eldh, AC; Tollne, A; Förberg, U; Wallin, L. | 2016 | What Registered Nurses Do and Do Not in the Management of Pediatric Peripheral Venous Catheters and Guidelines: Unpacking the Outcomes of Computer Reminders. | Include |
| 44 | Silva, JM; Stein, AT; Schünemann, HJ; Bordin, R; Kuchenbecker, R; de Lourdes Drachler, M | 2013 | Academic detailing and adherence to guidelines for Group B streptococci prenatal screening: a randomized controlled trial | Include |
| 45 | Stokes, T; Shaw, EJ; Camosso-Stefinovic, J; Imamura, M; Kanguru, L; Hussein, J. | 2016 | Barriers and enablers to guideline implementation strategies to improve obstetric care practice in low- and middle-income countries: a systematic review of qualitative evidence. | Include |
| 46 | Smith H; Ameh C; Roos N; Mathai M; Broek NVD. | 2017 | Implementing maternal death surveillance and response: a review of lessons from country case studies. | Include |
| 47 | O'Loughlin F; Phangmanixay S; Sisouk K; Phommanivong V; Phiahouaphanh O; Al Eryani S.; Raajimakers H; Gray A. | 2021 | Integrated Management of Neonatal and Childhood Illness Training in Lao PDR: A Pilot Study of an Adaptable Approach to Training and Supervision | Include |
| 48 | Trevisanuto, D; Marchetto, L; Arnolda, G; Chien, TD; Lincetto, O; Cavallin, F; Xuan, NM; Tien, NV; Hoi, NTX; Moccia, L. | 2015 | Neonatal resuscitation in Vietnam: a national survey of a middle-income country. | Include |
| 49 | Downe S; Finlayson K; Tunçalp Ö; Gülmezoglu AM | 2019 | Provision and uptake of routine antenatal services: a qualitative evidence synthesis. | Exclusion reason: Wrong population |
| 50 | Carter J; Sandall J; Shennan AH; Tribe RM | 2019 | Mobile phone apps for clinical decision support in pregnancy: a scoping review. | Exclusion reason: Wrong intervention |
| 51 | Mugambi ML; Pintye J; Heffron R; Barnabas RV; John-Stewart G | 2022 | HIV Prevention Tools Across the Pregnancy Continuum: What Works, What Does Not, and What Can We Do Differently? | Exclusion reason: Wrong study design |
| 52 | Chamberlain C; McNamara B; Williams ED; Yore D; Oldenburg B; Oats J; Eades S | 2013 | Diabetes in pregnancy among indigenous women in Australia, Canada, New Zealand and the United States. | Exclusion reason: Wrong outcomes |
| 53 | Myers KL | 2016 | Predictors of maternal vaccination in the United States: An integrative review of the literature. | Exclusion reason: Wrong outcomes |
| 54 | Dada S; Tunçalp Ö; Portela A; Barreix M; Gilmore B | 2021 | Community mobilization to strengthen support for appropriate and timely use of antenatal and postnatal care: A review of reviews. | Exclusion reason: Wrong intervention |
| 55 | Bhutani VK; Cline BK; Donaldson KM; Vreman HJ | 2011 | The need to implement effective phototherapy in resource-constrained settings. | Exclusion reason: Wrong study design |
| 56 | Yap D; Liang X; Garland SM; Hartley S; Gorelik A; Ogilvie G; Tan J; Wrede CDH; Jayasinghe Y | 2016 | Clinicians' attitude towards changes in Australian National Cervical Screening Program. | Exclusion reason: Wrong population |
| 57 | Lakiang T; Daniel SA; C KK; Horo M; Shumayla S; Mehra S | 2021 | Generating evidence on screening, diagnosis and management of non-communicable diseases during pregnancy; a scoping review of current gap and practice in India with a comparison of Asian context. | Exclusion reason: Wrong comparator |
| 58 | Heslehurst N; Crowe L; Robalino S; Sniehotta FF; McColl E; Rankin J | 2014 | Interventions to change maternity healthcare professionals' behaviours to promote weight-related support for obese pregnant women: a systematic review. | Exclusion reason: Wrong outcomes |
| 59 | Abadan SS; Hawryluk L; Montandon M; Flowers N; Schueller J; Eakle R; Patel P; Chevalier MS; Rana S; Amzel A | 2022 | Preexposure Prophylaxis Among Pregnant and Lactating People in 18 PEPFAR-Supported Countries: A Review of HIV Strategies and Guidelines. | Exclusion reason: Wrong study design |
| 60 | Peng Z; Wang S; Xu B; Wang W | 2017 | Barriers and enablers of the prevention of mother-to-child transmission of HIV/AIDS program in China: a systematic review and policy implications. | Exclusion reason: Wrong intervention |
| 61 | Melman S; Schoorel EN; Dirksen C; Kwee A; Smits L; de Boer F; Jonkers M; Woiski MD; Mol BW; Doornbos JP; Visser H; Huisjes AJ; Porath MM; Delemarre FM; Kuppens SM; Aardenburg R; Van Dooren IM; Vrouenraets FP; Lim FT; Kleiverda G; van der Salm PC; de Boer K; Sikkema MJ; Nijhuis JG; Hermens RP; Scheepers HC | 2013 | SIMPLE: implementation of recommendations from international evidence-based guidelines on caesarean sections in the Netherlands. Protocol for a controlled before and after study. | Exclusion reason: Study protocol |
| 62 | Ogunyemi DA; Fong A; Rad S; Fong S; Kjos SL | 2011 | Attitudes and practices of healthcare providers regarding gestational diabetes: results of a survey conducted at the 2010 meeting of the International Association of Diabetes in Pregnancy Study Group (IADPSG). | Exclusion reason: Wrong outcomes |
| 63 | Krawiec C; Mysore M; Mathur M; Fang X; Zhou S; Thomas N; Nakagawa T. | 2022 | Impact of the updated guideline for pediatric brain death determination on current practice | Exclusion reason: Conference abstract |
| 64 | Abdallah B; Whitford H; Bradbury-Jones, C; Jones M. | 2021 | Perceptions and attitudes of parents and healthcare professionals about the option of using infant massage in neonatal intensive care units | Exclusion reason: Wrong intervention |
| 65 | Kugelman, A. | 2019 | Neonatal pulmonology: "year in review" for the pediatric pulmonologist | Exclusion reason: Wrong study design |
| 66 | Davis AL; Carcillo JA; Aneja RK; Deymann AJ; Lin JC; Nguyen TC; Okhuysen-Cawley RS; Relvas MS; Rozenfeld RA; Skippen PW; Stojadinovic BJ; Williams EA; Yeh TS; Balamuth F; Brierley J; De Caen AR; Cheifetz IM; Choong K; Conway E; Cornell T; Doctor A; Dugas MA; Feldman JD; Fitzgerald JC; Flori HR; Fortenberry JD; Graciano AL; Greenwald BM; Hall MW; Han YY; Hernan LJ; Irazuzta JE; Iselin E; Van Der Jagt EW; Jeffries HE; Kache S; Katyal C; Kissoon N; Kon AA; Kutko MC; MacLaren G; Maul T; Mehta R; Odetola F; Parbuoni K; Paul R; Peters MJ; Ranjit S; Reuter-Rice KE; Schnitzler EJ; Scott HF; Torres A; Weingarten-Abrams J; Weiss SL; Zimmerman JJ; Zuckerberg AL. | 2017 | American College of Critical Care Medicine clinical practice parameters for hemodynamic support of pediatric and neonatal septic shock | Exclusion reason: Wrong outcomes |
| 67 | Olayemi E; Asare EV; Benneh-Akwasi Kuma AA. | 2017 | Guidelines in lower-middle income countries | Exclusion reason: Wrong setting |
| 68 | Martin SL; Omotayo MO; Pelto GH; Chapleau GM; Stoltzfus RJ; Dickin KL. | 2017 | Adherence-specific social support enhances adherence to calcium supplementation regimens among pregnant women | Exclusion reason: Wrong population |
| 69 | Tickell KD; Mangale DI; Tornberg-Belanger SN; Thitiri J; Timbwa M; Njirammadzi J; Chisti J; Ahmed T; Khan AF; Saleem AF; Kazi Z; Mupere E; Mukisa J; Sukhtankar P; Berkley JA; Walson JL; Denno DM. | 2017 | Barriers to pediatric inpatient care guideline adherence: A mixed method assessment of eight hospitals in Asia and Africa | Exclusion reason: Wrong population |
| 70 | Biringer A; McDonald S; Austin MP; Harvalik P; Van Zanten SV; Giallo R; Lasiuk G; McQueen G; Sword W; Vermeyden L; Kingston D. | 2017 | Mental health e-screening during pregnancy: Women's perceptions of risks and benefits | Exclusion reason: Publication not available |
| 71 | Rassi C; Gore-Langton G; Graham K; Gidudu B; Mufubenga P; Li M; King R; Siduda SG. | 2016 | Improving uptake of iptp in uganda through text messaging health workers | Exclusion reason: Conference abstract |
| 72 | Allard S; Barroso F; Pavord S; Spiby H; Khan K. | 2013 | Implementing new guidelines for the management of iron deficiency anaemia in pregnancy-incorporating the views of health care professionals and user groups | Exclusion reason: Conference abstract |
| 73 | Hermens RPMG; Van Den Boogaard E; Leschot NJ; Vollebergh JHA; Bernardus R; Kremer JAM; Van Der Veen F; Goddijn M. | 2010 | Poor adherence to the guideline on recurrent miscarriage: Identification of barriers | Exclusion reason: Conference abstract |
| 74 | Goodarzi, B; Seijmonsbergenâ-Schermers, A; Cronie, D; van Laerhoven, H; vanÂ den Akker, T; van Kaam, AH.; de Jonge, A. | 2023 | (Un)warranted variation in local hospital protocols for neonatal referral to the pediatrician: An explorative study in the Netherlands. | Exclusion reason: Wrong outcomes |
| 75 | Khanolkar AR; Hanley GE; Koupil I; Janssen PA. | 2020 | 2009 IOM guidelines for gestational weight gain: how well do they predict outcomes across ethnic groups? | Exclusion reason: Wrong outcomes |
| 76 | Sinclair R; Bajuk B; Guaran R; Challis D; Sheils J; Abdel-Latif ME; Hilder L; Wright IM; Oei JL; Abdel-Latif ME. | 2019 | Active care of infants born between 22 and 26Â weeks of gestation does not follow consensus expert recommendations. | Exclusion reason: Wrong outcomes |
| 77 | Wu M; Seel M; Britton S; Dean JA; Lazarou M; Safa Huda G; Paul G; Nourse C. | 2022 | Addressing the crisis of congenital syphilis: Key findings from an evaluation of the management of syphilis in pregnancy and the newborn in Southâ€East Queensland. | Exclusion reason: Wrong outcomes |
| 78 | Mustafa ST; Harding JE; Wall CR; Crowther CA. | 2022 | Adherence to Clinical Practice Guideline Recommendations in Women with Gestational Diabetes and Associations with Maternal and Infant Health: A Cohort Study. | Exclusion reason: Wrong population |
| 79 | Maruyama, N. | 2022 | American Academy of Pediatrics (AAP) Updated Infant Safe Sleep Guidelines Several Notable Additions. | Exclusion reason: Wrong study design |
| 80 | Keely, E. | 2012 | An opportunity not to be missed--how do we improve postpartum screening rates for women with gestational diabetes? | First round – Included. Consensus to exclude at data extraction and QA.  Exclusion reason: Wrong study design |
| 81 | Codipietro, L; Bailo, E; Nangeroni, M; Ponzone, A; Grazia, G. | 2011 | Analgesic Techniques in Minor Painful Procedures in Neonatal Units: A Survey in Northern Italy. | Exclusion reason: Wrong outcomes |
| 82 | Rhodes R; Smith Z; Adams J; Stoner J; Ali T. | 2014 | Assessment of Physicians' Perceived Risk of Inflammatory Bowel Disease Medications in Pregnant Patients. | Exclusion reason: Wrong outcomes |
| 83 | Long C; Quinonez R; Rozier R; Kranz A; Lee J. | 2014 | Barriers to pediatricians' adherence to American Academy of Pediatrics oral health referral guidelines: North Carolina general dentists' opinions. | Exclusion reason: Wrong population |
| 84 | Lioy J; Greubel E; Javia L; Soorikian L. | 2016 | Best practices for enhancing safety during high-risk bedside airway procedures in neonates. | Exclusion reason: Conference abstract |
| 85 | Omotayo M; Dickin K; O'Brien K; Neufeld L; De Regil L; Stoltzfus R. | 2016 | Calcium Supplementation to Prevent Preeclampsia: Translating Guidelines into Practice in Low-Income Countries. | Exclusion reason: Wrong population |
| 86 | McCarthy M; Glick R; Green J; Plummer K; Peters K; Johnsey L; Deluca C. | 2013 | Comfort First: an evaluation of a procedural pain management programme for children with cancer. | Exclusion reason: Wrong population |
| 87 | Tan M; Wright D; Syngelaki A; Akolekar R; Cicero S; Janga D; Singh M; Greco E; Wright A; Maclagan K; Poon L; Nicolaides K. | 2018 | Comparison of diagnostic accuracy of early screening for pre-eclampsia by NICE guidelines and a method combining maternal factors and biomarkers: results of SPREE. | Exclusion reason: Wrong outcomes |
| 88 | Mardegan V; Satariano I; Doglioni N; Criscoli G; Cavallin F; Gizzi C; Martano C; Ciralli F; Torielli F; Villani P; Fabio S; Quartulli L; Giannini L; Trevisanuto D. | 2016 | Delivery room management of extremely low birth weight infants in Italy: comparison between academic and non-academic birth centres. | Exclusion reason: Wrong outcomes |
| 89 | Sim C; Iida H; William F; Quinonez R; Steiner M; Vann W. | 2014 | Dietary recommendations for infants and toddlers among pediatric dentists in North Carolina. | Exclusion reason: Wrong population |
| 90 | Aldridge P; Rao A; Sethumadavan R; Briggs N. | 2018 | Fever under 3 months and the full septic screen: Time to think again? A retrospective cohort study at a tertiary-level paediatric hospital. | Exclusion reason: Wrong comparator |
| 91 | Stumbras, K; Rankin, K; Caskey, R; Haider, S; Handler, A. | 2016 | Guidelines and Interventions Related to the Postpartum Visit for Low-Risk Postpartum Women in High and Upper Middle Income Countries. | Exclusion reason: Wrong population |
| 92 | Geurts, DHF; Vos, W; Moll, HA; Oostenbrink, R. | 2014 | Impact analysis of an evidence-based guideline on diagnosis of urinary tract infection in infants and young children with unexplained fever. | Exclusion reason: Wrong population |
| 93 | Helou, A; Walker, S; Stewart, K; George, J. | 2017 | Management of pregnancies complicated by hypertensive disorders of pregnancy: Could we do better? | Exclusion reason: Wrong outcomes |
| 94 | Uppal, P; Cardamone, M; Webber, C; Briggs, N; Lawson, JA. | 2019 | Management of status epilepticus in children prior to medical retrieval: Deviations from the guidelines. | Exclusion reason: Wrong population |
| 95 | Reither, M; Germano, E; DeGrazia, M. | 2018 | Midwifery Management of Pregnant Women Who Are Obese. | Exclusion reason: Wrong intervention |
| 96 | Chessman, JC; Bowen, JR; Ford, JB. | 2017 | Neonatal exchange transfusions in tertiary and non-tertiary hospital settings, New South Wales, 2001-2012. | Exclusion reason: Wrong outcomes |
| 97 | Bartlow, KL; Cartwright, SB; Shefferly, EK. | 2016 | Nurses' Knowledge and Adherence To Sudden Infant Death Syndrome Prevention Guidelines. | Exclusion reason: Wrong outcomes |
| 98 | Mahmud, M; Mazza, D; Mahmud, M; Mazza, D. | 2010 | Preconception care of women with diabetes: a review of current guideline recommendations. | Exclusion reason: Wrong outcomes |
| 99 | Todd, FE; Roberg, Kathleen A; Welliver, RC. | 2010 | Preventing RSV Infection in At-risk Infants: Current and Emerging Strategies. | Exclusion reason: Wrong study design |
| 100 | Finocchario-Kessler, S; Clark, K; Khamadi, S; Gautney, B; Okoth, V; Goggin, K. | 2016 | Progress Toward Eliminating Mother to Child Transmission of HIV in Kenya: Review of Treatment Guideline Uptake and Pediatric Transmission at Four Government Hospitals Between 2010 and 2012. | Exclusion reason: Wrong comparator |
| 101 | Jebessa, S; Litch, JA; Senturia, K; Hailu, T; Kahsay, A; Kuti, KA; Wolka, E; Teklu, AM; Gezahegn, W. | 2021 | Qualitative Assessment of the Quality of Care for Preterm, Low Birth Weight, and Sick Newborns in Ethiopia. | Exclusion reason: Wrong intervention |
| 102 | Håkansson, S; Lilja, M; Jacobsson, B; Källén, K. | 2017 | Reduced incidence of neonatal early-onset group B streptococcal infection after promulgation of guidelines for risk-based intrapartum antibiotic prophylaxis in Sweden: analysis of a national population-based cohort. | Exclusion reason: Wrong outcomes |
| 103 | Tuohy, JF; Harding, JE; Crowther, CA; Bloomfield, FH. | 2019 | Reported adherence to current antenatal corticosteroid guidelines in Australia and New Zealand. | Exclusion reason: Wrong outcomes |
| 104 | Berisha, G; Boldingh, AM; Nakstad, B; Blakstad, EW;  Rønnestad, AE; Lee Solevåg, A. | 2023 | Retrospectively Assessed Muscle Tone and Skin Colour following Airway Suctioning in Video-Recorded Infants Receiving Delivery Room Positive Pressure Ventilation. | Exclusion reason: Wrong intervention |
| 105 | Gledhill, N; Scott, G; de Vries, NKS.; de Vries, NK. | 2018 | Routine follow-up of preterm infants in New Zealand. | Exclusion reason: Wrong population |
| 106 | Kreth, M; Shikany, T: Lenker, C; Troxler, RB. | 2017 | Safe Sleep Guideline Adherence in Nationwide Marketing of Infant Cribs and Products. | Exclusion reason: Wrong setting |
| 107 | Walker, D; DeMaria, LM.; Suarez, L.; Cragin, L. | 2012 | Skilled Birth Attendants in Mexico: How Does Care During Normal Birth by General Physicians, Obstetric Nurses, and Professional Midwives Compare With World Health Organization Evidence-Based Practice Guidelines? | Exclusion reason: Wrong outcomes |
| 108 | Riney, LC; Frey, TM; Fain, ET; Duma, EM; Bennett, BL; Murtagh Kurowski, E. | 2018 | Standardizing the Evaluation of Nonaccidental Trauma in a Large Pediatric Emergency Department. | Exclusion reason: Wrong population |
| 109 | Giglia, RC; Symons, M; Shaw, T. | 2019 | The provision of alcohol and breastfeeding information by maternal health practitioners in the Australian setting. | Exclusion reason: Wrong outcomes |
| 110 | NCT00344929 | 2006 | Severe Post Partum Haemorrhage (PPH): a Randomized Trial on Transversal Intervention in 6 French Perinatal Networks | Exclusion reason: Wrong publication year |
| 111 | Ignatov, P; Atanasov, B. | 2012 | Indirect standard cardiotocography plus fetal blood sampling versus indirect quantitative cardiotocography--a randomized comparative study in intrapartum monitoring | Exclusion reason: Full text not in English |
| 112 | Flenady, V; Gardener, G; Ellwood, D; Middleton, P; Boyle, F; Crowther, C; Coory, M; East, C; Callander, E; Gordon, A; et al. | 2017 | My Baby's Movements: a stepped-wedge, cluster-randomised controlled trial testing a mobile application intervention aimed at lowering stillbirth rates | Exclusion reason: Conference abstract |
| 113 | Nowe-Matheson, B; Kean, T. | 2014 | Implementing reminder strategies to increase rates of postpartum testing | Exclusion reason: Conference abstract |
| 114 | NCT02326207, | 2014 | Weekly Versus no Routine Ventilator Circuit Changes in NICU | Exclusion reason: Publication not available |
| 115 | Hauspurg, A; Sutton, EF; Catov, JM; Caritis, SN. | 2018 | Aspirin Effect on Adverse Pregnancy Outcomes Associated With Stage 1 Hypertension in a High-Risk Cohort | Exclusion reason: Wrong outcomes |
| 116 | Lawrence, A; Carson, V; Lewis, L; Swinbourne, A; Wicking, K. | 2018 | Relative advantage: findings from the optimising freedom of movement (OFM) study | Exclusion reason: Conference abstract |
| 117 | Maaloe, N; Housseine, N; Meguid, T; Saleh Khamis, R; Nielsen, BB; Bygbjerg, IC; Van Roosmalen, J. | 2018 | Effect of realistic childbirth guidelines (PARTOMA) on care and survival at Zanzibar's Main Hospital | Exclusion reason: Conference abstract |
| 118 | NCT04913584, | 2021 | Peer Administered Online CBT for PPD | Exclusion reason: Wrong outcomes |
| 119 | Kingsland, M; Doherty, E; Anderson, AE; Crooks, K; Tully, B; Tremain, D; Tsang, TW; Attia, J; Wolfenden, L; Dunlop, AJ; et al. | 2018 | A practice change intervention to improve antenatal care addressing alcohol consumption by women during pregnancy: research protocol for a randomised stepped-wedge cluster trial | Exclusion reason: Study protocol |
| 120 | ISRCTN23857995 | 2020 | Effects of electrical nerve stimulation during the first stage of labor | Exclusion reason: Wrong study design |
| 121 | NCT04211597 | 2019 | EGF-loaded Chitosan to Facilitate Healing and Prevent Scar Formation of Cesarean Wound | Exclusion reason: Wrong study design |
| 122 | Jabbour M; Newton AS; Johnson D; Curran JA. | 2018 | Defining barriers and enablers for clinical pathway implementation in complex clinical settings. | Exclusion reason: Wrong setting |

| **EXCLUSION CRITERIA KEY** | **DESCRIPTOR/S** |
| --- | --- |
| Wrong study design | Article is not a systematic review, qualitative, quantitative or mixed methods study. Projects reports and unpublished, grey literature. |
| Wrong setting | Setting other than maternity and neonatal (hospital, homebirth, birthing centre). |
| wrong population | Does not involve Healthcare professionals, Clinicians |
| wrong intervention | Does not involve Clinical recommendations, policies, clinical guideline, evidence based, policy |
| Wrong comparator | Does not concern barriers, enablers or facilitators |
| wrong outcomes | Does not involve Implementation, adoption, uptake |
| Conference abstract | Conference proceedings and abstracts |
| Study protocols | Study protocols without results and clinical trial registrations |
| Publication related | Not in English  Wrong year  Article not available |
