# Supplementary material for: Barriers and enablers for implementation of clinical practice guidelines in maternity and neonatal settings: A rapid review
Source: PLoS One. 2024 Dec 16;19(12):e0315588. doi: 10.1371/journal.pone.0315588 (PMC11649122; doi:10.1371/journal.pone.0315588)
Supplement: S2 File — (DOCX) [file pone.0315588.s002.docx]

**Supplementary File S2 Data extraction**

|  | **Author and year**  **Study origin/ country** | **Study type** | **Purpose/aim** | **Intervention (guideline, policy, recommendation)** | **Setting/ participants** | **Barriers / Enablers** | **Implementation approach / strategy** | **Outcomes/ results/conclusion** | **Limitations / Gaps** |
| --- | --- | --- | --- | --- | --- | --- | --- | --- | --- |
|  | Akuma 2012  United Kingdom (UK) | Quantitative Descriptive survey | To describe nurses and doctors’ knowledge and reported practice regarding procedural pain assessment and management in neonatal intensive care unit | Guideline (Association of Paediatric Anaesthetists of Great Britain and Northern Ireland 2008): application of knowledge of neonatal analgesia in routine practice | Doctors and nurses in seven NICUs in one area of UK | Interprofessional adherence to guideline (Nurses reported adhering to current guidelines (Association of Paediatric Anaesthetists of Great Britain and Northern Ireland 2008) more consistently than doctors. | not described, The study is a survey of clinicians' knowledge and application of guidelines (Association of Paediatric Anaesthetists of Great Britain and Northern Ireland 2008) . | Failure to relieve procedural pain in neonates is more widespread than previously suspected: depending on procedure, analgesia was not always administered by 45100% doctors and 5594% nurses. â€¢ Guidelines on assessing and minimizing pain in neonates are under-utilized by doctors, more so than nurses. â€¢ Clinicians are knowledgeable and aware of the hiatus between optimal and current practice | Associations inferred from survey results cannot be considered causal. |
|  | Albouy-Llaty et al. 2012  France | Quantitative Retrospective screening data analysis. | To evaluate guideline adherence among midwives and obstetricians related to perinatal Group B streptococcus (GBS) screening with process indicators. | Perinatal GBS screening guidelines. | Hospital data-AUDIPOG (Association des Utilisateurs de Dossiers Informatiss en PÃdiatrie, Obstétrique et Gynaecologic) database covering all French regions. Woman (n=5997) and neonates (n=84).  Midwives and obstetricians. | Barriers potential: Cost effectiveness and acceptability for screening  Enabler Clinician's ability to objective practice contributed to improved care.  Collaboration of clinicians in the goal of analysing the process and meticulously choosing valid and reliable indicators. Mandatory notification of new GBS data. Monitoring the defined indicators will facilitate assessment of improvement actions while promoting the appropriation of high-quality culture by care providers |  | Database entailed guideline adherence over a short lapse of time and resulted in a significant increase of the screening rate at the correct term.  However, circumstances where neonates are infected still remain.  Screening test performance needs to be reevaluated. |  |
|  | Alja'freh"and Abu-Shaikha 2021  Jordan | Quantitative cross sectional self-reported survey. | To assess the level of adherence of healthcare providers (HCPs) to Hypertensive Disorders (HDP) of Pregnancy guidelines (CPG) based on Health System Strengthening (HSS II) recommendations in Jordan. | Clinical practice guidelines (CPG) of HDP of Pregnancy. | Four hospital sites (public, private and military hospitals). Healthcare providers in maternal health services (n=270). Nurses, midwives, obstetricians. | Barriers: A lack of standardised evidence-based guidelines and clinical performance checklists to monitor and manage care. Lack of knowledge among nurses and midwives about the pathophysiology of the disease and their active role in monitoring the progress of mothers condition and appropriate management in order to decrease the risk of any complications and so, improve maternal and neonatal outcomes.  Enabler: Strengthening healthcare providers surveillance and clinical assessment capabilities to improve the pregnancy outcomes for the mother and her fetus. Educating nurses and midwives about the continued monitoring and reporting of unremitting symptoms in the postpartum period to minimize any possible complications. Nurses and midwives can do much in the administrative role in implementation. Nursing managers should develop and supervise the implementation of clinical guidelines to ensure that nurses and midwives understand and follow these guidelines and its evidence based recommendations and to periodically assess the effectiveness of such guidelines whenever possible.  Further attention should be given to provide sufficient resources to meet educational needs. Strategies to motivate healthcare providers and promote self-confidence in relation to clinical guidelines. Greater focus on continuous monitoring of professional conduct in regard to implementation of HDPs clinical guideline in different healthcare settings. |  | Results showed a suboptimal adherence of to evidence-based recommendations of HDPs guidelines; particularly in the antepartum and postpartum period.  Low from nurses and midwives adherence. Variations in obstetricians' practice. | Self-reported adherence. |
|  | Alsweiler et al. 2019  New Zealand | Quantitative Retrospective cohort study | To determine if the introduction of routine calculation of birthweight centiles in all infants improved adherence to a neonatal hypoglycaemia guideline and if adherence to the guideline was associated with the identification of neonatal hypoglycaemia in at-risk infants. | Neonatal hypoglycaemia guideline | Tertiary hospital. Maternal and neonatal data.  Infants (n=400) | Enablers: Routine use of calculated birthweight centiles was associated with improved adherence to the neonatal hypoglycaemia guideline and increased detection of neonatal hypoglycaemia in at-risk infants. Effective interventions that improved guideline adherence include periodic audits and the integration of guideline practice into the clinical environment. | Adherence audits and integration of guideline practice into the clinical environment. | Overall adherence was low.  Adherence improved from 2011 to 2015 (59/200 (30%) vs. 95/200 (48%), P < 0.001), with the largest improvement in large-for-gestational age infants (7/50 (14%) vs. 25/50 (50%), P = <0.001).  Screened infants whose care was adherent to the guideline had a higher incidence of hypoglycaemia detection (adherent, 64/154 (42%) vs. non-adherent, 34/246 (14%), P < 0.001). | Retrospective study focused on adherence to guidelines. |
|  | Breakell 2018  United Kingdom | Quantitative  Before and after audit  Quality improvement (QI) project. | To investigate the effect of implementing the bronchiolitis guideline, accompanied by an educational programme, on the frequency of use of CXR, antibiotics and nebulised treatment. | National Institute of Health and Care Excellence (NICE) bronchiolitis guideline | A District General Hospital in the North East of England (the education was targeted at clinical staff).  Clinical outcomes for 50 pre- and 51post-intervention patients. | Enabler: education | Audit of prior practice; education (including weekly briefings, posters, flow charts in clinical areas). (then prospective data collection post-implementation) | Implementation of the NICE bronchiolitis guideline, coupled with an educational program aimed at raising awareness, is very effective in reducing unnecessary CXR, antibiotic prescriptions and inhaled treatments in bronchiolitis. Overall NICE guideline compliance more than doubled, from 28 to 63%. | Small sample size; single centre. Use of retrospective data pre-intervention and prospective data collection (with resource limits to collection). |
|  | Brower et al. 2019  United States | Quantitative Retrospective baseline data evaluation of pre-and post-guideline intervention. | To increase the percentage of patients 0 to 60 days of age who are tested and treated for Herpes Simplex Virus (HSV) in accordance with local guideline recommendations from 40% to 80%. | Local guideline recommendation for treatment of HSV and use of acyclovir. | Free standing children's hospital (>650 beds) and satellite location (>40 beds).  0 to 60 days of age on the basis of local physician testing and treatment patterns for HSV. Six-month (Jan to June 2014) patient data (n=160) for pre-guideline, and 1120 clinical encounters with implementation. | Enablers: Point-of-care availability of an evidence-based guideline and interventions targeted at provider engagement improved adherence to a new guideline for neonatal HSV management and decreased acyclovir use in non-high-risk infants. | Paediatric care provider education (physicians and nurse practitioners) via presentations at conferences and divisional meetings.  Education for emergency department (ED) and intensive care nurses at conferences focusing on nursing care (complete laboratory values). Point-of-care guideline availability, copies of the flowchart posted in the medical staff workrooms.  Slideshow included in ED workspace screens.  Guideline added to electronic medical record (EMR) system allowing ED staff to access guideline directly from patient's chart for just in time reminders at point of care. A new provider order set developed in the EMR for the evaluation of serious infection in infants with subgroups based on age and HSV risk status.  New order set and subgroups modification in EMR to support clinical management according to guideline recommendations. Downward shift in adherence (resident changeover phase/new academic year) addressed through re-education for all frontline providers. | The median percentage of patients managed according to guideline recommendations increased from 40% to 80% within 8 months. Acyclovir use decreased from 26% to 7.9% (P , .001) in nonhigh-risk patients but did not change significantly in high-risk patients (73%83%; P 5 .15). There were no cases of delayed acyclovir initiation in HSV-positive cases. | Study limited to one setting limits generalisability.  Different methods used to study pre- and pot-intervention adherence cohort, impact on difference not known. |
|  | Brozanski et al. 2020  United States | Quantitative  Quality improvement project. | To describe the development and implementation of a standardised process to maintain perioperative euthermia. | Perioperative euthermia clinical practice recommendations | Children's hospital NICUs (n=19) from a Children's Hospital Neonatal Consortium (CHNC). Preoperative paediatric and neonatal healthcare professionals (physicians, surgeons, anaesthesiologists, nurses) | Barriers: Differences related to contextual factors within an organization that includes the microsystem, macrosystem, and the external environment.  Variation in QI capability across centres and culture at local level may impacted outcomes. Negative outcomes despite compliance to recommended process can impede buy-in (adoption) Variations in process, resources, practices at individual centres or across sites.  Enablers: Interdisciplinary engagement. Team learning and sharing through monthly webinar meetings (barriers and successes), bimonthly huddle teleconference (discussed project topics), active email group communications, and coaching by quality improvement (QI) advisors.  Short educational sessions to promote QI knowledge for participants. Continuous compliance monitoring and data collection through shared data repository. Award best sites/teams (best data records and compliance), promote friendly competition and collaborative learning). Top performing teams reported ability to actively engage and motivate an interdisciplinary team of stakeholders at all levels led to both system and process change.  Ability for teams to collaborate and make continuous process improvement. | Collaborative stakeholder (interdisciplinary paediatric and neonatal leaders from CHNC centres) engagement to define best local clinical processes related to maintaining perioperative euthermia. Used local Plan-Do-Study-Act (DSA) cycles to stimulate learning and collaboration. The CHNC collaborative did not dictate a specific bundle.  Participating sites expected to select those interventions that best fit their local site resources, talents, and needs. Each centre developed their own system for measuring compliance (most common strategy checklist/monitoring form that accompanied patient. Shared data repository to track and graph local data over time and monitor improvement progress and team assessment scores. | Postoperative hypothermia decreased by 48%, from a baseline of 20.3% (January 2011 to September 2013) to 10.5% by June 2015.  Strategies associated with decreased hypothermia include .90% compliance with patient euthermia (36.137.9Â°C) at times of OR arrival (odds ratio: 0.58; 95% confidence interval [CI]: 0.430.79; P , .001) and OR departure (odds ratio:0.0.73; 95% CI: 0.560.95; P = .017) and prewarming the OR ambient temperature to .74Â°F (odds ratio: 0.78; 95% CI: 0.620.999; P = .05).  Hyperthermia increased from a baseline of 1.1% to 2.2% during the project.  No thermal burns were reported. | Contextual differences and variation in QI capability across centres may have impacted centre outcomes. |
|  | David et al. 2021  Zimbabwe | Mixed methods study with retrospective medical record data and clinician interviews. | To model and identify gaps requiring service and care delivery improvement in prenatal care pathways for pregnant women visiting a central hospital. | Prenatal care pathways for pregnant women. | Hospital.  Maternity case records (n=100) and Interviews (n-20) with maternity care clinicians (nurses, midwives, gynaecologists, obstetricians). | Barriers: Cost factors - booking fee and cost of travel to hospital (against a background of poverty, low resources, long distance to travel). Economic factors - low resource country. Lack of effective ways to remind woman about ongoing appointments. Use of risky alternative faith-based care - cultural influences. Absence of resources to deliver care. Shortage of specialised maternity care staff and antenatal trained clinicians.  Paper-based documentation and lack of electronic tracking system.  Inadequate methods of health promotion (lectures and individual discussion were inadequate).  Challenges with pathway itself- haphazard nature of the health care delivery system.  Enablers: An investment into prenatal care by the government to enable the utilisation of interventions such as e-health technologies that may improve care delivery as well as adherence to best practices.  E-health and mobile health technologies involving e-referrals, e-booking, decision support, Electronic decision support and reminder systems for clinicians to manage and deliver appropriate care to patients as well as pregnant women to adhere to scheduled visits. e-booking and e-referral system that allows several functionalities such as tracking of referrals, appointments and automated reminders to women. Electronic or telecommunication system such as telephone, SMS, MMS or WhatsApp messaging application to remind women to attend appointments. Improve health promotion effort through electronic /online information delivery channels. More orderly and traceable pathway, streamlining between primary and secondary care to avoid congestion and inappropriate referrals for prenatal care. Buy-in and monitoring from the top-level Ministry of Health and Child Care,  Reduced booking costs for women with limited resources. |  | There were inadequacies in the care delivered to women in each visit compared to that expected in such areas as obstetrics, physical examinations and haematological tests.  The current prenatal care pathway at MCH requires improvement in the areas of referral, adherence to appointment by pregnant women and visiting prenatal care early. Clinicians also need to adhere to standard clinical tests recommended for each specific pregnant womans visit. |  |
|  | de Silva Carvalho et al. 2021  Brazil | Mixed methods- An exploratory case study and content analysis to qualify the composition of the corpus and descriptive statistics. | To synthesise contributions of the Brazilian public (women, health professionals, managers, educational institutions, and companies) about the implementation of the National Clinical Guidelines for Care in Normal Birth from the public consultation carried out in Brazil. | National Clinical Guidelines for Care in Normal Birth. | Public health system | Barriers: Associated with lack of financial resources. Lack of training and professional motivation. Resistance from the government and political disinterest. Structure of the health system and the professionals involved. Lack of humanized care. Poor facilities at hospitals. Lack of human resources and materials as well  An overburdened national health system. Resistance to change on the part of health professionals  Lack of financial support for maternity hospitals and the municipal care model.  Enablers for guideline implementation Obstetric nurses emerged as an alternative for supervising normal births. Mandatory presence of an obstetrician during childbirth in hospital settings. Training to change the practices of health professionals. Training and changing medical and hospital protocols. Awareness among health professionals and greater collaboration from management.  Increasing professional training particularly obstetric nurses. Training dissemination and awareness among patients, family members and health professionals about the need for change. |  | Five classes emerged from text analysis: mandatory presence of an obstetrician during labor and delivery in hospital settings; barriers and facilitators for guideline implementation; use of evidencebased practices by health professionals; progression of labor and delivery and womens rights; and mobilization to promote the guideline. | Only analyses texts (corpus). |
|  | de Oliveira Carvalho 2013  Brazil | Qualitative  Interviews | To verify the acting of the nursing team, with the puerperas, facing the process of breastfeeding and prevention of  difficulties of the mother breastfeeding, in rooming-in care of a reference maternity in the county of Juazeiro do Norte, CE, Brazil | Intervention is assuming the responsibility expected:  The nursing team of Rooming-in care and the Human Milk Bank have the responsibility of transmitting the maternal breastfeeding clinical management of lactation and the  orientation/techniques to prevent early difficulties during  breastfeeding | Childrens Friend Hospital, Brazil  8 nurses (7 nurse technicians; 1 nurse) involved in supporting breastfeeding. | Barrier:  Lack of health teaching  Lack of knowledge (Mothers need orientation to breastfeeding prenatally)    Enablers:  Need strategies to prevent problems with lactation. | by default, it is the expectation that the nurses will orient the mothers to breastfeeding during pregnancy and help to prevent lactation problems as part of their responsibilities. | Content analysis of interviews: of relevance here: prevention of problems with lactation and orientation to mothers varied between staff. | none specified |
|  | Doherty et al. 2020  Australia | Quantitative Randomised stepped-wedge controlled trial. Self-reported online/telephone interview questionnaire. | To estimate the effectiveness of a practice change intervention in improving the provision of antenatal care addressing alcohol consumption during pregnancy in public maternity services. | Introduction of recommended model of care addressing alcohol consumption during pregnancy based on systematic review of evidence, international and Australian clinical guidelines. | Public maternity services, antenatal care.  Three sectors (one urban, two regional/rural) of a single local health district in New South Wales.  Hospital and community-based midwifery clinics; hospital medical clinics; midwifery continuity of care group practices; Aboriginal Maternal and Infant Health Services (AMIHS); and specialist services caring for women with complex pregnancies or social vulnerabilities. Pregnant woman (n=5694). | Enablers: Model of care was acceptable which may have contributed to adoption of practice change intervention by clinicians. | Implementation involved a staged process.  Barriers and enablers to implementation first explored, including knowledge and resources to support practice.  Implementation strategies incorporated targeted/necessary behaviour change techniques.  Application of selected implementation strategies developed through stakeholder consultation (key antenatal providers and managers) in each sector. Content and delivery of implementation strategies tailored to each sector's usual processes.  Cultural appropriateness embedded into the implementation strategies through consultations with Aboriginal health staff, local community members and organisations, as well as focus groups with Aboriginal women who had recently attended a participating maternity service. | The intervention was effective in increasing womens reported receipt of: assessment of alcohol consumption (OR: 2.63; 95% CI: 2.263.05; p <0.001), advice not to consume alcohol during pregnancy and of potential risks (OR: 2.07; 95% CI: 1.782.41; p <0.001), complete care relevant to alcohol risk level (advice and referral) (OR: 2.10; 95% CI: 1.802.44; p<0.001) and all guideline elements relevant to alcohol risk level (assessment, advice and referral) (OR: 2.32; 95% CI: 1.942.76; p<0.001).  Greater intervention effects were found at the 2728 and 3536weeks gestation visits compared with the initial antenatal visit. No differences by sector were found. Almost all women (98.8%) reported  that the model of care was acceptable. |  |
|  | Eldh 2016  Sweden | Qualitative (nonparticipant observation and interviews) | To depict if, in what context, and how computer reminders regarding evidence-based management of peripheral venous catheters (PVC) in paediatric care are applied according to RNs actions and experience. | Computer reminders about PVC clinical practice guidelines | Pediatric university hospital with approximately 245 beds in 16 units (including emergency, medical, surgical, orthopedic, NICU), employing approximately 940 RNs altogether | Barrier:  Alert-reminder design /interface  Duration of reminders were too short | Computer reminders at the time of reading the electronic patient record at the beginning of shift. At NICU level, PVCs were recorded in a template | Computer reminders, designed to improve adherence, did not really work.  NICU: 3 out of 4 RNs noticed the computer reminders if they used the PVC template; none if the template was not used.  RNs who recognised the reminders suggested that they were displayed for too short a time to be acknowledged.  Clinical practice (nonparticipant observation) mainly followed best practice (with variations). Although acknowledged, reminders were not considered helpful. | Provides supplementary findings regarding how a single intervention can work and in what context.  Due to patients at the time, could not include all intervention units, thus limiting understanding of varied contextual factors. |
|  | Gkentzi et al. 2017  United Kingdom | Systematic review | To summarise and present the current knowledge on antenatal vaccination against pertussis with regard to national recommendations, coverage, immunogenicity, safety and effectiveness of the current available vaccines. | Recommendations for national immunisation program- antenatal vaccination against pertussis. | Literature in English from January 2011 to May 2016  with searches in four databases. | Low uptake factors: Barriers (contextual factors- country-location impact) USA-Young maternal age, absence of public insurance, ethnicity/race and premature delivery. Mexico-Most popular reason for refusal was concern about vaccine safety. Korea-Women of child-bearing age appeared not to be adequately informed by healthcare providers (HCPs) about the vaccinations recommended in pregnancy. Germany -migration status was associated with lower vaccine uptake in pregnant women. USA-obstetric provider concerns about vaccine safety and efficacy, despite the growing body of literature against these beliefs. USA-Logistics and financial barriers-vaccine price, storage, inventory and inadequate reimbursement, the need to vaccinate in each pregnancy, vaccine safety and low incidence of pertussis in the area.  Enablers:  Mexico-Recommendation of vaccination by an obstetrician was a strong determinant of vaccine uptake. UK-Multiparous women were more likely to accept antenatal vaccinations including pertussis and to participate in vaccine trials during pregnancy compared with primiparous. UK- HCPs were the most important and valuable source of information for pregnant women. UK - antenatal care and vaccination provided mainly by general practitioners in primary care (costs, reimbursement, vaccine inventory). Australia- new mothers were more likely to be vaccinated if they had heard of the disease and the benefits of vaccination from an HCP. | Proven-strategies to increase uptake: Electronic-phone-based best-practice antenatal alerts reminding HCPs to vaccinate pregnant women under their care from 32 weeks gestation and at each subsequent encounter up to delivery until vaccination is performed and recorded. Implementation of a hospital-based outpatient pertussis prevention clinic. Better education of the public and healthcare professionals. | 47 studies reviewed. To achieve high vaccination coverage in countries with national recommendations - educational programs were needed to improve provider vaccine confidence and recommendation and public education.  Assistance to overcome financial and logistics barriers. |  |
|  | Gu et al. 2020  China | Quantitative, controlled before-and-after-implementation study. | To describe the implementation process of a nutrition risk screening and assessment guideline for infants with congenital heart disease and to assess the impact of nurses behaviour and the effect on infants outcomes. | Implementation of an Evidence-Based Guideline of Enteral Nutrition for Infants with Congenital Heart  Disease. | Cardiac center at Childrens Hospital of Fudan University, Shanghai, China.  Infants (n=142) with congenital heart disease and nurses (n=100). | Barriers  Innovation (guideline development) - Guideline is not available in an accessible and usable form; Guideline is perceived as beyond the scope of nursing duties. Recipients (nurses) -Lack of knowledge and skills of nutrition risk screening and nutrition assessment  in infants with CHD; Not considering nutrition risk screening and nutrition assessment as important; Not conducting nutritional risk screening accurately; Not completing nutritional assessment for infants independently; Limited communication between doctors and nurses about the nutritional care of infants. Context (cardiac centre) - Lack of human resources, such as clinical dietitians; Lack of multidisciplinary cooperation mechanism and working processes; Involvement in department affairs decision-making is not high; Lack of incentives (job prospects, learning opportunities, remuneration, personal honors, etc.).  Enablers  Understanding nurses' attitude and behaviours pre-implementation.  Positive attitude toward acquisition of new knowledge, acceptance of change to clinical practice. Implementation of evidence-based guideline to support clinical practice. Targeted action plan - Knowledge training, tools improvement, skills training, redistribution of clinical roles, update of hospital information system (HIS) and establishing communication and feedback processes to share information. | Duration of implementation process was four months.  Developed several specific action plans for identified barriers, including knowledge training, tools improvement, skills training, clinical roles redistributing, Hospital Information System (HIS) update, and establishing communication and feedback processes to share information. | Knowledge, attitude, and behavior of nurses about nutrition risk screening and assessment increased significantly after implementing the guideline. Nurses compliance with the recommendations for nutritional risk screening improved significantly on three criteria; assessment of nutritional status stability (p < 0.001), assessment of nutritional status deterioration (p = 0.003), and nutritional assessment among  infants with moderate risk and above (p < 0.001). The nurses compliance with the recommendations for nutrition assessment improved significantly in eight of the 10 criteria (p < 0.001).  The proportion of infants receiving comprehensive nutrition assessment when they were first screened with moderate or high nutritional risk were higher in the intervention group (24.3% vs 83.3%; p < 0.001). The accuracy rates of nutrition risk screening were higher in the intervention group (52.9% vs 81.9%; p < 0.001). | Short duration of implementation timeframe. Impact of contextual factors, including other health disciplines, patient factors, observation-based studies. Limited to one setting/centre. |
|  | Haskell et al. 2021  New Zealand and Australia | Mixed methods study Quantitative component measured intervention fidelity.  Qualitative component examined clinical leads perceptions of the interventions via an online questionnaire. | To determine whether the interventions were implemented as planned (fidelity), explore end-users perceptions of the interventions and evaluate cluster randomised control trial (cRCT) outcome data with intervention fidelity data. | Targeted theory-informed interventions to improve bronchiolitis management in acute paediatric setting | 26 hospitals in Australia and New Zealand.  Emergency and paediatric inpatient units. Intervention (n=13) and control (n=13). Clinicians and clinical leads (nursing and medical). | Barriers Challenges of time constraints greatest barrier for use.  Enablers  Championed via clinical leads. Stakeholder meetings. Train the trainer workshops. Delivery of education for staff during implementation. Use of other educational materials (videos, fact sheets, posters). Monthly audit and feedback on action plan and uptake. | Roll-out over six months, during the Australian and New Zealand bronchiolitis season, 1st May 2017 to 30th November 2017. Intervention hospitals received theory-informed interventions targeting nursing and medical clinicians who managed infants with bronchiolitis in the ED and paediatric inpatient units. Control hospitals received an electronic and printed copy of the complete Australasian Bronchiolitis Guideline and interventions at the completion of the study. Interventions developed using a stepped theory-informed approach - Five evidence-based recommendations from guideline; identified factors perceived to influence treatment; factors mapped to behaviour change techniques most likely to effect change for identified factors; targeted interventions mapped to behaviour change techniques based on feasibility, local relevance, and acceptability of the interventions. Consistent sequence of interventions across sites. | The cRCT found targeted, theory-informed interventions improved bronchiolitis management by 14.1%.  The process evaluation data found variability in how the intervention was delivered at the cluster and individual level.  Total fidelity scores ranged from 55 to 98% across intervention hospitals (mean=78%; SD=13%).  Fidelity scores were highest for use of clinical leads (mean=98%; SD=7%), and lowest for use of other educational materials (mean=65%; SD=19%) and audit and feedback (mean=65%; SD=20%).  Clinical leads reflected positively about the interventions, with time constraints being the greatest barrier to their use. |  |
|  | Kebaya et al. 2018  Kenya | Quantitative. Retrospective baseline audit of medical records and follow-up questionnaire. | To assess compliance with evidence-based criteria regarding newborn resuscitation among healthcare workers in the maternity units in a District Hospital. | Evidence-based criteria regarding newborn resuscitation. | Maternity units in a busy district hospital.  Healthcare providers working in labor ward, maternity theater, postnatal and newborn units. | Barriers  Resistance to change. Lack of incentives/ motivation to attend training and meetings. Poor collaboration among maternity nurses and between maternity and paediatric units. Knowledge gaps amongst healthcare providers. Time constraints to provide education and training due to availability of staff, trainers and busy units.  Staff workloads.  Enablers Regular meetings and discussions promoting evidence-based practices. Identifying change champions. Incentives to increase attendance to meetings and training. Enhanced teamwork through joint unit meetings. Assigning roles and delegating tasks to specific people. Printed and audio-visual education resources and equipment for skills training provided by hospital. Acknowledging input motivated staff. Flexible training designed around scheduled staff work hours or during shift changeovers. | Evidence-based audit criteria were developed based on an evidence summary developed by JBI.  Used the JBI PACES and Getting Research into Practice (GRiP) audit and feedback tool.  Implementation framework involved three phases of activity- Establishing project team representing unit staff and clinicians; reflecting on results of baseline audit and designing and implementing targeted strategies to address non-compliance (barriers); and conducting follow-up audit to assess outcomes of interventions to improve practice and potential future issues to be addressed subsequently. | Project was successful in improving awareness, knowledge and skills on basic newborn resuscitation to health care providers. All staff received training basic newborn resuscitation. The project improved the quality and access to equipment in the facility as the audit was able to highlight the availability or absence of some of this equipment. Protocols updated related to basic newborn resuscitation and equipment. | Limited time of project restricted further evaluation. |
|  | Langley et al. 2015  Canada | Quantitative cross sectional survey | To determine existing infection prevention and control practices related to MRSA and additional precautions (AP) and other practices in Canadian paediatric healthcare settings. | Canadian Nosocomial Infection Surveillance Program (CNISP). Methicillin-Resistant Staphylococcus aureus (MRSA) transmission in pediatric health care facilities. | 50 hospitals. Infection control professionals working in settings providing care to children in Canada. | Barriers: Lack of agreement about which measures should be used in settings that provide care to infants, children and youth and their families. Presence of multiple guidelines to prevent nosocomial spread of antibiotic-resistant organisms (ARO) in general, and MRSA specifically resulting in variation. Lack of consistency and variations in recommended practice within different guideline documents. Focus on need to facilitate removal of AP in the long stay patient, clinician preference, repeated infections, or in the setting of an outbreak. Lack of evidence-based guideline recommendations impacting understanding and compliance of health care providers.  Enablers: More consistent and evidence based guidelines for the pediatric setting could improve confidence in recommendations and hence compliance with them. |  | Although policies and procedures to prevent MRSA transmission in the health care setting are routine in Canadian paediatric settings, there is variation in application of national guidelines. Evidence-based guidelines specific to the care of infants, children and youth would help improve consistency cross care setÂ­tings, and understanding and compliance with infection prevention and control policy by health care providers, patients and families. | Limited to survey results. Lack of qualitative data from provider |
|  | Laubscher et al. 2013  Switzerland | Quantitative, questionnaire. | To evaluate acceptance of Swiss guidelines to prevent vitamin K deficiency bleeding (VKDB) by paediatricians of the Swiss Society of Paediatrics. | Swiss guidelines to prevent vitamin K deficiency bleeding (VKDB). | Paediatricians of the Swiss Society of Paediatrics (n=629) | Barrier  Refusal of prophylaxis treatment by parents. |  | The main risk factors for VKDB in breast-fed infants are parental VK prophylaxis refusal or an unknown cholestasis. | Narrow study focus. |
|  | Luitjes et al. 2018  Netherlands | Quantitative  Cluster randomised control trial (cRCT) | To evaluate the effectiveness of an innovative strategy to improve implementation of evidence-based guidelines on the management of hypertension in pregnancy compared to a common strategy of professional audit and feedback. | Obstetric guidelines on the management of hypertension in pregnancy. | Hospitals providing obstetric care (n=16). Health professionals - obstetricians and gynaecologists, residents and clinical midwives. Patients- pregnant women with hypertensive disorder. | Factors- Reasons for not using DSS (barriers to change) Limited use of DSS in practice. Not integrated into daily routine or clinical workflow in electronic records. Professionals either were quite indifferent to an innovative strategy type or did not consider it their job to use the DSS.  Enabler Automated embedded system of reminders, therapeutic suggestions and diagnosis specific links to guidelines could be more effective. | Compared two implementation strategies. Innovative multifaceted implementation strategy including a computerized decision support system (DSS) and professional audit and feedback reports, and meeting. Minimal implementation strategy of audit and feedback reports only. | No statistically significant difference was found in both the occurrence of major complications and most secondary outcome measures between the two groups.  Innovative strategy increased guideline adherence in one item- laboratory testing. Process evaluation showed limited use of the computerized DSS, with a large variation between hospitals (049,5% of the eligible patients), but positive experiences of actual users. Using a computerized DSS for implementation of the clinical guidelines for the management of hypertension in pregnancy did not result in fewer major maternal and foetal complications.  Limited use of the DSS in the innovative strategy group could be an explanation for the lack of effect. | Study did not achieve statistical power. |
|  | Lyngstad et al. 2021  Norway | Quantitative  Quality improvement (QI) project. | To evaluate implemented guidelines for pain assessment and management and increased parental involvement in a Norwegian single-family room NICU. | Guidelines for pain assessment and management and increased parental involvement in single-family room NICU. | NICU, interprofessional clinical staff, and parents | Enablers Theoretical lectures on the subject of non-pharmacological interventions and parental involvement in  procedures. Practical bedside supervision 24/7 to interprofessional staff increased parental involvement in stress- and painful procedures. Daily reflections to the interprofessional staff during the implementation. Focus on ergonomic equipment (flexible chairs and tables) to facilitate parental involvement and make it more practical and comfortable for parents and staff.  Cultural change agents (champions) for QI project. Staff involvement in QI project from onset and positive attitude and commitment. Pain assessment and flowcharts integrated into current routines and existing workflows (avoid extra workload). | Stepwise approach based on a Plan-Do-Study-Act (PDSA) framework. Plan a desired change, execute the elements of the change (do), study the change by measured outcomes, and then adjust on the gained information (act) to begin another cycle. Steps - assessment of problem; literature review; development of pain management strategy; education of NICU staff; implementation of new pain tool and guidelines; monitoring of quality improvement data; and continuous improvement.  Development and implementation of guidelines with flowcharts for pain management, and pain assessment certification of the interprofessional staff. | One year after implementation 88.8% of pain assessments were performed according to the pain management guidelines.  Compliance with guidelines increased. Staff systematically used flowcharts to assess, treat and reassess pain and stress. Showed increased parental involvement in procedures, from 50.3% before to 82.3% after the quality improvement project. Increased the nurses and doctors awareness of procedural pain and the importance of parental involvement. | Limited to staff compliance. Did not assess impact of intervention on infant population. |
|  | Mohan et al. 2023  United States | Quantitative retrospective study. | To evaluate the use of intravenous immunoglobulin (IVIG) in infants with haemolytic disease, to assess compliance with the American Academy of Paediatrics (AAP) guideline recommendations, and to review the data on which the guidelines were based. | American Academy of Paediatrics (AAP) guideline recommendations for intravenous immunoglobulin (IVIG) in infants with haemolytic disease. | Hospital, NICU (82 beds), infants. | Potential barriers to compliance Lack of understanding of the possible side effects, cost, and donor exposure related to IVIG. Lack of awareness of current recommendations for practice.  Enablers Education and clarification on current evidence for practitioners. Education around new/current clinical practice guideline. Development of explicit local (institution) protocol based on APP criteria and recommendations. | Development of explicit local institutional guideline based on APP criteria and recommendations (evidence-based practice). Education and clarification around current practice guidelines for practitioners. | Most infants who received IVIG for hyperbilirubinemia did not meet the AAP criteria, prompting development of an institution-specific IVIG clinical practice guideline.  The 2022 AAP guideline was published after this study was completed, but it confirmed belief that IVIG usage should be more restricted and the criteria more explicit. | Single centre study. Small sample. Retrospective nature of study limited collection of baseline pre-admission data. |
|  | Moore et al. 2020  Canada | Qualitative interview and content analysis | To identify facilitators and barriers to implementation of a clinical practice guideline developed to support shared decision-making for these cases. | Guideline supporting shared decision making for extreme preterm birth. | Children's' Hospital. Healthcare Care Provider (HCP) (16 physicians, nine nurses). | Barriers included: (1) minimal awareness or familiarity with the guideline content; (2) lack of agreement with the recommendations; (3) inadequate evidence and applicability to support changes in practice; and, (4) lack of resources to care for the most immature infants (organisational constraints).  Enablers  to implementation included: (1) an awareness of, familiarity with and belief in the content; (2) hard copy and electronic guideline accessibility; and, (3) institutional expertise to provide necessary care. | Proposed- based on findings (to overcome identified barriers and inevitable resistance to change from various groups of HCP) 1) improvement in the pre-implementation blueprint to minimize knowledge gaps and broaden stakeholder input;  2) support for HCP in terms of continued education, case debriefings and adequate resource provision; and,  3) more extensive training, with a greater focus on the supportive evidence and philosophical and ethical arguments demonstrating how shared decision making takes into consideration the diverse attitudes and opinions regarding the care options for EPI. | Identified facilitators and barriers can inform the development of tailored strategies for improved local and future broader implementation. | Single institution. Limited to health workers perceptions (barriers and enablers). |
|  | Muhumuza et al. 2015  Uganda | Quantitative  Pre-post (audit) | To evaluate hand hygiene compliance among health care workers in the paediatric special care unit. | Hand hygiene to reduce transmission of health care worker-associated pathogens. | Paediatric special care unit of a national referral hospital. Health care workers (HCW). | Barriers Workload and overcrowding. Staff attitude and lack of knowledge about evidence-based practice. Environment factors- lack of resources/equipment (lack of easy access to clean water and inadequate cleaning equipment). Contextual factors - limited priority afforded to educating staff.  Enablers Education sessions for staff raising awareness about best practice and infection potential. Distribution of materials describing best hygiene practice (posters and brochures) and reminders (posters) for staff. Equipment distribution by providing hand cleaning equipment at strategic locations (five moments of hand hygiene). Increasing awareness among staff and patients (what to expect from HCW) | Three phases including- baseline audit, implementation of best practice strategies and follow-up audit. Joanna Briggs Institute the Practical Application of Clinical Evidence System (JBI PACE) online audit tool and Getting Research into Practice (GRiP) framework to guide project processes.  Three strategies staff education and awareness, staff sensitisation and reminders, and provision of infection control equipment (hand cleaning equipment) were implemented to overcome identified barriers. | Compliance with best practice hygiene was found to be poor in the baseline audit for all but one of the audit criteria.  Following the implementation of the strategies, hand hygiene improved.  The compliance rate increased substantially across all criteria.  Staff education achieved 100%, whilst criterion 4 increased to 70%.  However, use of alcohol-based hand-rub for hand hygiene only improved to 66%, and for six of the seven audit criteria, compliance remained below 74%. | Single centre study. Short timeframe of implementation, lack of project sustainability. |
|  | Muirhead and Kynoch 2019  Australia | Quantitative Pre/post audit design | Evidence implementation project to improve pain assessment and management in neonates receiving an opioid infusion, and to prevent or decrease the incidence of iatrogenic withdrawal syndrome (IWS). | Evidence-based clinical guideline for the management of neonatal pain published by the Australian New Zealand Neonatal Network. | Two intensive care areas of a Neonatal Critical Care Unit (NCCU) of a large tertiary referral hospital (79 beds). | Barriers (1) The pain tool forms utilized in the clinical area were not formatted to allow for accurate documentation of nonpharmacological measures. (2) Lack of staff awareness concerning the degree of poor compliance to pain assessment in the NCCU. (3) Insufficient knowledge of the incidence and severity of IWS occurrence in the NCCU. (4) The large cohort of staff. (5) Time restraints and heavy workload. (6) Inconsistency in practice.  Practice change  Enablers Review and update of current policy, pain assessment tools and forms to align with best practice recommendations. Dissemination of baseline audit results (displayed in unit, emailed, unit meetings). Education package developed (PowerPoints presentations emailed and made available electronically to unit staff). Role modelling best practice to prompt behaviour (practice change)  Interdisciplinary collaboration. Visual summary protocol prompts/reminders integrated with bedside charts improve consistency of practice and adherence. Scoring tools based on a set of objective physiological clinical signs and symptoms (vs subjective indicators. | Development of a weaning protocol based on clinical guideline for the management of neonatal pain implemented along with targeted staff education to align current practice with best practice recommendations. Implementation of strategies targeting identified barriers pre-implementation. Development of evidence-based practice change strategies to improve clinical care of neonates on opioid infusions in the NCCU informed by pre-implementation audit results. Team leaders and clinical nurses appointed with task of role modelling best practice to prompt behaviour (practice change).  Laminated instruction and protocol summary cards were also created and added to the bedside chart as another adjunct to assist in improving consistency of practice and adherence. | Results demonstrated an improvement in the completion of pain assessments by 34%, and 100% compliance to withdrawal assessment following the introduction of an IWS assessment tool.  For neonates receiving analgesics for less than 4 days, adherence to the weaning schedule occurred in 75%. No clinical signs of IWS were seen in this cohort.  For neonates receiving analgesics for greater than 4 days, adherence to the weaning schedule occurred in only 55%. Of those neonates where the protocol was not followed, 67% developed clinical signs of IWS. Lack of adherence to the pain management policy and weaning tool led to increased awareness of the importance of collaboration within the multidisciplinary team to improve outcomes. Consistency of practice and change-management remain a challenge in clinical care. | Small sample size, Single centre study. Impact of acuity of patient factors on results. Baseline staff knowledge not assessed. |
|  | Nair et al. 2014  United Kingdom | Systematic literature review. | To conduct a global situational analysis to identify the current facilitators and barriers to improving quality of care (QoC) for pregnant women, newborns and children. | Quality of care |  | Enablers and barriers to improving QoC for pregnant women, newborns and children identified in this metareview can be grouped into the six strategic management domains of the WHO’s framework (information, patient and population engagement, regulations and standard, leadership, organisational capacity and models of care).  Need to focus on user-provider communication and satisfaction. | Proposed- based on findings  1. Health systems should set minimum standards of communication between users and providers, and also, among providers across the various levels of the healthcare system to improve effectiveness and efficiency of care provision. 2. Language barriers, especially among the migrant and minority population, could be addressed with the help of qualified interpreters. Where necessary, health systems should recruit and train a cadre of qualified interpreters with effective communication skills. This would be particularly important considering the inequities and disparities in care provision faced by certain sections of the population in all countries. 3. Training on communication skills should be incorporated as an integral part of health professionals education. Regular training of providers could also be an effective means of addressing several issues related to healthcare provision through improving the technical and communication skills, competence, confidence, cultural sensitivity, attitude and behaviour of providers. However, it ought to be acknowledged that training without an enabling environment will not improve the overall health outcomes and QoC. 4. CHWs have proven to be effective in mobilising and engaging users and communities in several LMICs. It is important for health systems to train and retain this valuable resource. 5. Midwives were found to be a valuable resource in HICs, and services provided by them were as effective and acceptable as that provided by doctors. This resource should be utilised well in LMICs by building their capacity through training and supportive supervision. 6. Audit and feedback, and training of providers are important strategies to improve adherence to regulations and standards. However, efforts are required to address the implementation challenges and methodological issues of audit and feedback to achieve. greater benefits | Limited to gathering evidence from systematic reviews and meta-analyses. | It is about barriers and facilitators for quality of care. |
|  | Nkamba et al. 2017  Zambia and Democratic Republic of Congo (DRC) | Qualitative, group interviews and focus groups. | To identify context-specific barriers and facilitators to the implementation of antenatal screening and treatment during pregnancy. | Implementation of antenatal screening and treatment during pregnancy. | Primary care clinics (n=11) Clinic administrators, health care workers (midwives, nurses, physicians, lab technicians, counsellors and nutritionists), and pregnant woman (n=112). | Barriers a) System level: Structural constraints (beyond healthcare system), fragmentation of the health system, existence of Antenatal Care (ANC) guidelines in conflict with proposed intervention, poor accessibility of clinics (geographical and functional), staff and product shortages at the PCCs.  b) Healthcare providers level: lack of knowledge and training about evolving best practices, reservations regarding same-day screening and treatment.  c) Pregnant women level: late enrolment in ANC, lack of knowledge about consequences and treatment of syphilis, stigma, and need for partner consent.  Enablers Existence of national guidelines for ANC best practice recommendations (including testing and counselling at first visit). Public health initiatives to improve quality of care. Recognition of importance of early treatment. Willingness to improve methods for testing. Recognized importance of ANC visits Health education provided at the clinics. Acceptance of same-day screening and treatment at first ANC visit. | Recommendations for the design of the Preventive Congenital Syphilis Trial (PCS Trial) intervention based on targeted strategies to overcome identified barriers (context-specific factors). Implementation components-  Ensuring supplies needed for testing. Behavioural intentions - select opinion leaders (peer nomination) to disseminate information. Reminders targeting health professionals (written, print- posters). Audit and feedback - data monitoring and evaluation (robust data registries and data analysis). Women counselling - short/simple messages and printed illustrated information in local languages for pregnant woman (low literacy). Plan and tailor intervention according to context of implementation. Supportive supervision for health professionals during implementation. Creation of clinical workflow charts to guide health professionals' practice.  Including implementers and other end-users in intervention design essential to developing interventions that will be both effective and feasible (Co-design). | The final intervention was informed by implementers understanding of what would work in the field. Components included in the intervention were considered acceptable and feasible for implementation at the PCCs. | Specific to context. Small study. Self-reported, potential bias. |
|  | O'Loughlin et al. 2021  Lao | Quantitative  Pre- and post-training survey. | To evaluate pilot implementation of redesigned paediatric care training and determine whether the new approach was feasible and acceptable to health workers and if health centers where staff have participated in a new model of training showed improvements in pediatric case management. | Integrated management of neonatal and childhood illness (IMNCI) guidelines- national strategy. | Provincial-and district-level hospital staff (n=56) and health centre workers (nurses, midwives and healthcare assistants (n=43). | Barriers: Lack of resources (essential medicines). IMNCI trained staff encounter difficulties upskilling their colleagues in specific skills (e.g., differentiating between wheeze and stridor) or in meeting standards of documentation because of time constraints or low skill level in this area.  Enablers: Training was valued, active participation and engagement. Skills gaps addressed through further practice during supervision visits. Education techniques - Interactive, allow for practice and feedback, high degree of interaction with the educator, utilize multiple teaching techniques including group problem-solving. Cascade model-of Inservice training suitable for low-resource settings with the ability to train a large number of participants quickly. | Redesigned training to use multimedia tools. Removed long facilitator led-training sessions. Flexible coaching style modules to suit training needs and different learning strategies. Teaching activities were learner centred, active learning, capability building and structured to facilitate transition from theory to practice. Training program implemented in three progressive stages to develop capacity and capability and train large number of staff in low resource environments. Participants completed pre-post evaluation on training. | Participants participated actively during the training and that the majority were confident in performing individual steps of IMNCI. Improvement between pre-and post-training scores in case management. In-service education programs that keep adult learning principles at their core can result in improvements in healthcare worker action. |  |
|  | Olsen et al. 2018  United States | Quantitative  Quality Improvement (QI) project- retrospective data analysed. | To develop standardised nutritional guidelines that would promote increased growth velocity (GV) in premature infants. | Nutritional guidelines for premature infants. | Children's hospital -neonatal intensive care unit (NICU) (74-beds).  Interdisciplinary NICU team staff. | Enabler: Vital information was delivered in multiple ways to support various learning styles.  Electronic communications with guidelines attached alerted staff to upcoming changes.  Presentations provided all disciplines with updated materials. Printed guidelines were placed in bedside notebooks for NNPs and nurses.  For electronic access, guidelines appeared on our internal Web-based system, which were continuously available to all staff.  For quick access, the residual algorithm was laminated and secured to every bedside computer screen.  Education plan was completed before the scheduled go-live date. Providers were allowed to deviate from the  guidelines with proper documentation in the medical record. Clinicians -staff champions to promote guideline adherence. | Standardised QI technique, the Edwards Demings Plan Do Study Act (PDSA) change model. An evidence-based literature review and development of a feeding advancement guideline. NICU staff voted on guideline acceptance and adoption. Staff education on the guidelines in a multipronged  approach with a planned go-live date. Clinical practice/care (total parenteral nutrition (TPN)) data collected and monitored during the implementation period.  Four cycles of PDSA with data review and improvement through education, reminders and updates/modification to guideline. | Compliance with feeding guidelines quickly improved and was maintained at about 70 percent at the conclusion. Successful implementation of guidelines and standardisation of practice.  Well educational initiative was an important factor that promoted the project. | Single centre study. |
|  | Page et al. 2017  Australia | Qualitative, semi structured interviews and content analysis | To identify the barriers to the delivery of early optimal nutrition in a tertiary-level Neonatal Critical Care Unit to inform implementation strategies in translational research to ensure best practice care. | Nutrition guidelines for infants who weigh <1500 gms (preterm birth). | Neonatal Critical Care Unit (NCCU) in a tertiary-level hospital. Medical (Consultants, Fellows and Registrars) and nursing (experienced, recent graduates and Clinical Nurse Facilitators) staff from the NCCU (n=19). | Eight barrier domains were identified using Theoretical Domains Framework (TDF). -Knowledge; memory, attention and decision processes; skills; professional/social role and identity; beliefs about capabilities; beliefs about consequences; environmental context and resources; and social influences.  Enablers Researchers who are clinicians within the unit being aware of champions and points of leverage for targeting when initiating changes Support from the hospital and unit management for evidence-based practice/implementation of projects, Positive audit and improvement culture in the unit Good intention of the staff to provide good nutritional care to the units babies. | Proposed implementation strategy (based on identified barriers) Consensus on guidelines  Develop nutrition bundles and link with other guidelines. Redesign work processes to enhance adoption. Leadership, awareness, championing and promotion. Incentivisation and enablement  Parental nutrition solutions and streamlined ordering. Education and modelling initiatives.  Persuasion and ongoing communications during transition. | Four main themes, with associated sub-themes identified: (i) Roles and responsibilities, (ii) decision making, (iii) disconnect between beliefs and the application of evidence and (iv) monitoring and awareness. Bridge the evidencepractice gap by targeting implementation strategies to affect behaviour change. | Single location. |
|  | Pangerl et al. 2021  Australia | Systematic literature review | To provide a synthesis of what is known about compliance with Group B Streptococcus (GBS) screening protocols in a variety of global settings, including maternity homes, private obstetric practice, and hospital clinical environments. | Group B Streptococcus Screening Guidelines in Pregnancy. | Studies located in maternity homes, private obstetric practice, and hospital clinical environments. | Barriers (lack of adherence reasons) Lack of awareness amongst midwives of consequences of GBS. An assumption of the test procedure being complicated. Avoidance of collaboration with the hospital obstetricians. Low compliance in private practice due to financial factor- not covered by health insurance (out of pocket expense). High proportion of caesarean sections and GBS swabs not indicated due to low risk of GBS transmission with caesarean section births.   Enablers Effectiveness of GBS vaccines. Education about the importance of the prevention of GBS disease via a variety of formats to achieve higher compliance rates with GBS screening guidelines. Academic detailing to enhance clinical decision making, utilising nonbiased information using a variety of methods for evidence-based care and better patient outcomes. Computer-generated or electronic alert or reminder systems. Forming response teams and creating a clinical pathway. Establishing ownership of and responsibility for clinical protocols. Creative and interactive multifarious staff education and periodic evaluation of compliance with feedback. Quality important screening program reducing need for repeated screening. Multilingual literature about GBS for pregnant women to assist with decision making. |  | Six studies from different countries focused on adherence to GBS screening guidelines were reviewed.  Different factors such as financial aspects and high caesarean section rate may have an influence on adherence to GBS screening protocols. Implementation of strategies and different forms of education can result in improved compliance rates. | Lack of qualitative information. |
|  | Pricilla et al. 2018  Kenya | Quantitative Retrospective study of programmatic data. | To assess progress made in adopting prevention of mother to child transmission of HIV (PMTCT) guidelines and associated outcomes. | World Health Organisation's (WHO) Prevention of mother to child transmission of HIV (PMTCT) treatment guidelines. | Four government hospitals in Kenya. Mother-infant pairs (n=2604) | Barriers Missed diagnosis if infants cannot be traced for new samples. Need for diagnostic tools and algorithms to evolve along with updated guidelines. Point of care processes. Lack of data or missing data - poor records.  Enabler Strengthening strategies to improve monitoring and support for medication adherence and retention in care. | National level adoption. Implementation at facility level. | Results indicated positive trends in adopting latest PMTCT guidelines by the four hospitals in Kenya. | Generalisability limited as the study only looked at mother-infant pairs enrolled in the HIV infant tracking system. |
|  | Rousseau et al. 2020  France | Mixed methods. Before and after cross-sectional vignette-based survey study. | To describe spontaneous preterm birth prevention practices self-reported before and after the dissemination of relevant guidelines, and to identify personal and organisational factors associated with adherence. | National guidelines for obstetrics. | French obstetricians practicing in public or private maternity units.  Before n=286, after n=282, and both n=145. | Barriers  Quantitative analysis factors in non-adherence - older age and practice in non-university or small hospitals (organisational factors- status of units).  Qualitative analysis for barriers to adoption - fear of change, individual and organisational practices and habits, individual beliefs, questions over legitimacy /reliability of guidelines, work overload, lack of time, complexity, lack of clarity and applicability of guidelines, guideline awareness, availability and accessibility, insufficient communication or restricted dissemination.  Enablers (modifiable) Related to the format of the guidelines, such as the use of keywords or a decision tree, and to their wider multi-professional dissemination through regional networks, maternity units, distribution by e-mail or the internet. |  | Adherence to guidelines was generally low, with practices unmodified by their dissemination.  No improvement was observed after dissemination. Improvement is required, especially regarding applicability. |  |
|  | Ryan et al. 2020  United States | Systematic review | To synthesis data about implementation outcomes of obstetric haemorrhage (OH) prevention innovations in low- and middle-income countries (LMIC). | Postpartum haemorrhage (PPH) clinical guidelines, policy and management of obstetric haemorrhage (OH) prevention. | Obstetrics, LMICs | Barriers Challenges among intervention beneficiaries, such as pervasive myths/fears of uterotonics and other sociocultural barriers. Challenges among providers (i.e. intervention implementers), including issues with knowledge, awareness and use of evidence-based interventions. Challenges from the health system, including supply chain bottlenecks and human resource limitations.  Successes - Enablers Successful delivery linked to targeted implementation strategies (Chaudoir et al., 2013; Procter et al., 2011): focused on Implementation outcomes - Acceptability, adoption, appropriateness (fit), costs, feasibility, fidelity, penetration, and sustainability.  Service outcomes - Efficiency, safety, effectiveness, equity, patient centeredness, timeliness. Patient outcomes - Obstetric haemorrhage incidence and severity. | Development and testing of implementation strategies. Early community engagement in value of intervention. Stakeholder engagement in designing delivery (co-design). Use of a theory of change that clarifies and guides how intervention activities lead to outcomes. Continuous shared learning for quality improvement Training and monitoring (multimodal, including simulation, peer learning) Utilization of reminders to use evidence-based interventions. Use of opinion leaders.  Guidance by external mentors.  Early advocacy efforts at the state level for national policy implementation. Monitoring data collection systems | Various barriers were reported, including challenges among intervention beneficiaries, providers and within the health system; however, studies reported the development and testing of practical implementation solutions. These included training and monitoring of implementers, community and stakeholder engagement and guidance by external mentors.  Some studies linked successful delivery to implementation outcomes, most commonly adoption and acceptability, but also feasibility, penetration and sustainability. Findings suggest that innovations to prevent OH can be acceptable, appropriate and feasible in LMIC settings; however, more research is needed to better evaluate these and other under-reported implementation outcomes. | limited to review of databases. |
|  | Sharma et al. 2021  Norway | Qualitative  Focus groups with purposive sample. | To advance the knowledge regarding the mechanisms behind suboptimal follow-up in the Nordic and South Asian women with previous gestational diabetes mellitus (GDM) by comparing (1) their experiences, (2) health and disease perceptions and (3) barriers to and facilitators of health-promoting behaviours. | Lifestyle-changes guidelines essential for preventing diabetes post-GDM. | Three hospital outpatient clinics. Woman 1-3 years after a pregnancy with GDM (n=23) | Non-adherence: Barriers Women's real-life constraints. Lack of more organised public healthcare during and after GDM. |  | Five main themes were identified: lack of resilience, emotional distress, â€˜caught between a rock and a hard place, postpartum abandonment and insufficient guidance.  Key determinants of the maintenance of unwanted health behaviours after GDM were consistent across the ethnic groups.  Recommendation to promote specific coping strategies and changing the healthcare service approach rather than relying on women's capacity to initiate the necessary changes. | Small study. Single location. |
|  | Silva et al. 2013  Brazil | Quantitative Randomised controlled trial (RCT). Before and after intervention. | To examine effectiveness of academic detailing (AD) of obstetricians, compared with clinical practice guidelines (CPG) direct mail and no intervention, on the screening of pregnant women for Group B Streptococcus (GBS) | Guidelines for Group B streptococci prenatal screening. | Medical cooperative. Prenatal outpatient care-community. Obstetricians (n=241) | Barriers Lack of additional education.  Enablers Academic detailing along with direct mail printed guidelines. Education provided by trained physician. | Intervention period three months. The AD group received the guidelines and an educational visit detailing the guidelines conducted by a trained physician.  The direct mail group were only sent guidelines on GBS. | Women cared for by obstetricians who received an AD- based intervention were more likely to be screened for GBS than were those cared for by obstetricians who received printed guidelines only or no intervention. | Small sample size. Low power. Loss of participants during study. |
|  | Skare et al. 2018  Norway | Quantitative  Pre-post test | To evaluate whether neonatal resuscitations skills and team performance would improve after implementation of video-assisted, performance-focused debriefings. | Neonatal resuscitations skills | University hospital delivery wards.  Compared 74 resuscitation events pre-implementation to 45 events post-implementation. Midwives and physicians. | Adherence  Barriers: Initiating resuscitation without paediatrician (physician) being present at the beginning  Enablers NRPE-score was higher when paediatrician (physician) was present from the beginning of resuscitation.  Debriefing after the event and reflective practice.  Educational approach to debrief. | A modified Neonatal Resuscitation Performance Evaluation (NRPE) was used to score team performance and procedural skills during a 7-month study period (2.5, 2.5 and 2 months pre-, peri- and post-implementation) (median score with 95% confidence interval). Video-assisted performance focused debriefing. Debriefings scheduled as soon as possible after the event. | Implementation of video-assisted, performance- focused debriefings improved adherence to best practice guidelines for neonatal resuscitation skill and team performance. Technical and non-technical skills improved after intervention. Technical skills had greatest improvement. | Single institution study. Small sample. Limited to pre-post-test, no qualitative data. |
|  | Smith et al. 2017  United Kingdom | Qualitative  Case studies - secondary analysis | Review of a series of country case studies in order to determine whether and to what extent these countries have implemented the four essential components of maternal death surveillance and response (MDSR) and identify lessons for improving implementation. | Implementing maternal death surveillance and response (MDSR) and Maternal Death Review (MDR) systems. | 10 case studies -countries as part of World Health Organisation (WHO) Global MDSR Implementation survey. 10 countries at different stages of implementation of Maternal Death Review (MDR) systems. | Barriers  Lack of planning around implementation and monitoring of responses and health system challenges. Low/poor budget allocations for health and low prioritisation and poor planning. Lack of sustainable resources (financial and human). Lack of trained health workers to identify and collect data. Poor coordination and information flow between faculty and district or central committees. Poor quality of reporting, inaccurate reporting, poor data collection, use of handwritten reports (illegible and insufficient information. Lack of supervision or monitoring of reporting process (weak registration systems). Lack of leadership, knowledge, training   Enablers Policy level- Strong government commitment and involvement. Enforcement of MDR policies by professional bodies/colleges. Adequate legal framework. Use of review of data to assign funding, and revise performance indicators. District level- Accurate data collection (data quality) Electronic systems for rapid assessment and analysis. systematic identification and dissemination of remedial actions Recommendations targeted at different levels of health system. Facility level-Commitment of unpaid health professionals participating as part of professional development. |  | Overall, although MDR is at least introduced, accepted and ongoing at subnational level in many countries, it is not institutionalised at national and subnational level and the shift from facility based MDR to continuous MDSR that informs the wider health system still needs to be made. | Selected countries reported on. |
|  | Snelgrove-Clarke et al. 2015  Canada | Quantitative Randomised controlled trial. | To determine the effects of an Action Learning intervention on nurses use of a fetal health surveil lance (FHS) guideline during labor of women who were low risk on admission for delivery. | Fetal Health Surveillance (FHS) Guideline in Clinical Practice. | Regional tertiary care hospital, perinatal centre. Birthing unit. Nurses (n=89). | Factors impacting adherence Barriers: (Clinical indicators) Nurses identified events during labor such as signs of a nonreassuring fetal heart rate (abnormal), as the dominant type of inhibitor to the uptake of the guideline.  Nurses identified nonreassuring fetal heart rate and the use of an epidural analgesia as the main situations that led them to do continuous electronic fetal monitoring rather than intermittent auscultation. Inconsistency among colleagues, deeply entrenched or long-standing practice and lack of education, and values and beliefs impacted adoption.  Enabler: Practice environment. Availability of equipment. Clear unit policy. Clinical indicators impacting clinical decision-making (signs) | Groups of four to six nurses participated in monthly, 2-hour Action Learning meetings (first introductory meeting 4 hours).  Six months period. | Statistically significant change was not evident between nurses rate of FHS practices in the Action Learning group compared with Usual Care (6.8%, odds ratio [OR] 0.16, 95% confidence interval [CI] 0.842.83). Despite lack of between-group significant changes in FHS practices, Action Learning nurses, who chose areas of practice that presented obstacles to their guideline adherence ability (epidurals and narcotics), significantly changed their FHS practices. | Single site study. Small sample. Underpowered. |
|  | Stokes et al. 2016  New Zealand | Systematic review | To synthesise qualitative evidence on guideline implementation strategies to improve obstetric care practice in low- and middle-income countries (LMIC) in order to identify barriers and enablers to their successful implementation. | Guidelines to improve obstetric care practice. | Low- and middle-income countries (LMIC)- Sub-Saharan Africa and in hospital obstetric health care facilities. | Barriers: were themed under  Pre-implementation phase, implementation phase and  Institutionalisation phase. Professional motivation influenced all stages.  Specific barriers: Poor recording and data quality. Inconsistency in data collection - various/numerous workers. non-motivated data collection. Audit meetings were blaming exercise- culture. No local clinical leadership. Audit meetings were uni-professional (dominated by traditional medical hierarchies). Poor communication of audit meeting findings and feedback. Lack of feedback and recommendations communicated across the health system.   Enablers: Good recording and extraction of clinical information. Experienced, qualified, and trained data collectors. Audit meetings as a learning exercise. Local clinical leadership. Multidisciplinary audit meetings. Good communication of audit findings and feedback. Communicating findings and recommendations across the health system. | Two guideline implementation strategies identified:  Clinical audit and feedback (both criterion-based audit and maternal death reviews). Educational interventions. Propose a model: (See Figure 2) Address high and low intrinsic professional motivation. Target changes in stages  Pre-implementation - create awareness and commit to implement. Implementation- prepare to implement and implement. Institutionalisation - integrate into routine practice and sustain integrated practices. | Nine studies included. Identified a number of quality improvement processes that are amenable to change at limited or no additional cost, although some identified barriers may be difficult to address without increased resources. Applied a stages of change model to explain results. professional motivation was also key to change. Pathways to implementation were complex and understanding of individual and organisational behaviours and motivation in LMIC settings needs more research. | Limited database search. |
|  | Sundercombe et al. 2014  Australia and New Zealand. | Quantitative  Cross-sectional survey and guidelines quality appraisal (using checklist) | To establish how well postnatal ward neonatal hypoglycaemia guidelines facilitate breast feeding and adhere to UNICEF UK Baby Friendly Initiative (BFI) recommendations, and to compare compliance with different recommendations. | Postnatal ward neonatal hypoglycaemia guidelines and UNICEF UK Baby Friendly Initiative (BFI) recommendations. | Tertiary neonatal centres (n=23) in Australia and New Zealand. | Barriers  quality: Lack of up-to-date references or flowcharts.  Lack of evidence-based recommendations. Variation in recommendations across guidelines. Complexity of guidelines and dated, unreviewed versions.  enabler: Auditing and practice review. User friendly and up to date guidelines. Evidence-based recommendations. |  | Guideline quality appraisal generally scored poorly. Compliance with the recommendation to promote skin-to-skin contact and early breast feeding was poor across all centres.  Few guidelines included up-to-date references or flowcharts.  Guidelines suggest necessary screening for neonates. Guidelines need constant revision as evidence for best practice expands. | Small sample. Limited to specific context. Did not examine reasons for low adherence. |
|  | Telfer et al. 2021  United States. | Quantitative  Chart audits and root-cause analysis. | To implement an evidence-based bundle at an urban community teaching hospital in at least 50% of labors in 60 days in order to reduce early labor admissions and increase adherence to evidence-based labor manage- ment guidelines shown to decrease cesarean birth. | Evidence-Based Bundle to Reduce Early Labor Admissions | Teaching hospital birthing unit. Team- certified nurse-midwife, obstetrician, resident, student, nurse-midwife, pediatric provider, scrub technician, three to four nurses, and an anesthesia team. | Barriers Initial staff resistance to change- loss of autonomy. Use of checklists (paper-based) varied among staff.  Enablers: Patient experience survey- felt birth choices respected, shared decision making in labour support and coping techniques. Patient education - instructional handouts. Use of point of care tools/checklists to drive guideline adherence.  Audit and feedback. Team engagement.  Recommendations: integrate checklist into routine EMR workflow.  Design patient surveys for electronic handheld device use for ease of use and data analysis. | An early labour triage guide - education, checklist pilot and modification, posters, patient education. Labour walking path- Introduced and polit tested, modified, progressive staff involvement and families.  Audit and feedback -partograph, and pre-caesarean checklist. Communication and feedback on findings, education to reduce variations, integrated into EMR. Staff engagement- change of shift huddles, group and one-on-one education sessions, monthly meetings, manager rounds, goal reminders via email communications, small groups, celebrating milestones. Patient experience survey. Four Rapid Cycle Plan Do Study Act (PDSA) cycles were conducted over 8 weeks for accelerating QI initiative. | The bundle was implemented in 58% of births. The bundle reduced early labor admissions labor from 41% to 25%.  Team knowledge reflecting current guidelines in labor management increased 35% and 100% of cesareans for labor arrest met criteria.  Patient satisfaction scores exceeded 98%. Implementing an evidenced-based bundle was effective in reducing early labor admissions and increasing utilizationof and adherence to labor management guideline. |  |
|  | Trevisanuto et al. 2015  Vietnam | Quantitative Survey | To evaluate the consistency of resuscitation practices, and adherence to the international guidelines for neonatal resuscitation, in a large representative sample of hospitals in Vietnam. | International guidelines for neonatal resuscitation. | Public central, provincial and district hospitals (n=160) representing the three levels of public hospital-based maternity services. | Barriers Lack of essential equipment. Knowledge deficit. Lack of training updates. Different levels of maternity services resulted in variation in practices affecting quality of care. Lack of resources - low resource settings (middle-income country)  Background of professional or clinical leader involved in management of the newborn at birth. Existence of different protocols, and international vs national guidelines. Guideline-recommendation variations among protocols.  Enablers: Consistency in policy.  Additional training and updates. Standardisation of guidelines and recommendations. | Proposed: Simple low-cost interventions could be implemented to improve neonatal outcomes, particularly in district hospitals where a large proportion of the births in Vietnam take place. | Neonatal resuscitation was provided by obstetricians and, or, midwives at all levels.  Half of the hospitals did not follow recommendations for delaying cord clamping.  Significant variations in resuscitation practices between central, provincial and district hospitals and limited adherence to international recommendations. |  |
|  | Turan et al. 2012  Kenya | Quantitative  Cluster randomised controlled trial. | To evaluate the effects of integrating HIV treatment into Antenatal Care (ANC) clinics at government health facilities in rural Kenya. | HIV and Antenatal Care Integration in Pregnancy in Kenya | Rural government health facilities (n=12). Pregnant HIV-positive woman. | Barriers:  Implementation challenges Implementation delays and changes in services environments. Changes in multiple national guidelines during implementation period. Lack of critical clinic and laboratory supplies. Inadequate transportation systems to deliver supplies. High staff turnover and shortages. Movement-reassignment of staff between services. Diversity of facilities and population served. Geographical locations- large distances to travel and difficulties with follow-up care.  Enablers Staff training. Ongoing refresher training. Data collection and monitoring. Monitoring of data collection process- form completion. Cost-effective model. | Staff training and ongoing updates. Internal monitoring of form completion and quality of data entry on monthly basis.  Use of database. Monitoring of clinics on service delivery and aspects on service integration. | Baseline results revealed that the intervention and control cohorts were similar with respect to socio- demographics, male partner HIV testing, seroiscordance of the couple, obstetric history, baseline CD4 count, and WHO Stage.  Challenges faced while conducting this trial at low-resource rural health facilities included frequent staff turnover, stock-outs of essential supplies, transportation challenges, and changes in national guidelines. |  |
|  | Trollope et al. 2018  New Zealand | Quantitative  Self-administered questionnaire. Appraisal of Guidelines Research & Evaluation (AGREE) II instrument standards used to assess adherence. | To explore the reasons for poor compliance with maternity guidelines. | Maternity clinical practice guidelines (n=10) developed by National Womens Health. | 10 purposively selected guidelines related to maternal and neonatal care. Clinicians (n=82). | Barriers Extent of stakeholder involvement, rigour of development, applicability, editorial independence. Different guidelines used to inform practice (national and international). Clinicians' perceptions - Reduces autonomy of practitioner, not up to date with current evidence-based medicine, denies individuality of patient, guideline recommendations will not lead to desired outcomes in care, difficulty to locate (accessibility), authors are biased, cannot follow recommendations because clinician lacks confidence or preparation, oversimplified guidelines, and not applicable to practice (patient) population.  Enabler: Clarity of presentation. Clinicians' positive perception of guidelines -worthwhile and useful to guide practice, aid decision making, incorporate new research, improve patient outcomes, benchmark for standard clinical practice, reduce variations in practice.  Encouragement from senior staff members and peers would encourage their use.  Monitor performance and provide feedback. Introduce sanctions is not used. Link payment/ reimbursement to guideline use. |  | Poor guideline development may have contributed to failure of the local maternity guidelines, it appears that accessibility is a major barrier to their use and implementation. |  |
|  | Warren 2011  Australia | Pre- and post-implementation audit strategy. | To determine nurses understanding and management of infants with intravenous (IV) therapy. | Protocol for the prevention and management of extravasation injuries in the neonatal intensive care. | Neonatal critical care unit (NCCU) (79-cots) of a large tertiary level hospital. | Barriers: No recent education.  Knowledge deficit among different levels of nursing staff (senior-junior) Resistance to change from senior nurses to relocate or rotate to other areas with less experienced staff to support the junior staff.   Enablers: Protocol in place. Initial and ongoing staff education and increased awareness. High impact location of information for staff (educational boards displayed in nurses' station) Audit and feedback of findings.  One-on-one staff feedback and discussion. Mentor and support junior staff. | Evidence-informed protocol development - literature review. Joanna Briggs Institute (JBI) Getting Research into Practice (GRIP) program.  Audit, feedback and re-audit sequence to enhance change and improve clinical practice.  Project was implemented in four stages over a 7-month period over six months. | Initially, there was poor compliance with all four criteria, ranging from zero to 63%.  Following implementation of best practice, the second audit showed a marked improvement in all four criteria, ranging from 70 to 100% compliance. The GRIP phase of the project identified five barriers which were addressed throughout this project.  Overall, this project has led to improvements in clinical practice in line with current evidence. This has resulted in enhanced awareness of the risks associated with IV therapy and of measures to prevent an injury occurring within this clinical setting. | Limited to single discipline in the unit.  Recommend  in future use of online training and videos to support staff education - new medical and nursing. |
|  | Wilkinson et al. 2017  Australia | Quantitative  Cross-sectional prospective online survey. | To re-assess staff knowledge, attitudes and behaviours around the management of gestational weight gain (GWG) following service changes at a maternity hospital through a second (Time 2) survey. | Clinical guidelines regarding weight management in pregnancy- best practice delivery of care to pregnant women regarding GWG. | Tertiary maternity hospital. Antenatal staff (obstetricians, midwives and allied health) (n=69) | Barriers: Lack of awareness and knowledge of current Local state-based guideline. Relied on alternative National, Professional peak body guidelines and clinical speciality policy. Variation in referral practices. Variable practice opinions of staff. Lack of readily available resources (e.g., weigh scales). Lack of time.  Enablers: Readily available guidelines. More staff training and willingness to participate in training.  Having sufficient resources available to support women. Staff believing that women find their advice helpful. Woman having resources and motivation to effect behaviour change. Sufficient dietetic referrals available. active hospital-based service improvement and research culture at clinical and managerial levels. Availability of specialist maternal health dietitians and physiotherapists (allied health staff). | Propose strategies/recommendations for future based on study: Use service-wide policy approach in management of GWG (guideline implementation). Operationalise interventions through skills-based training, engagement of clinical champions, recording of weight (data) at agreed millstones and data monitoring, and availability of additional resources (e.g., extra weigh scales in consulting rooms). Focus on skill development and behaviour change. Include health consumer in practice implementation. | Varied adoption of guidelines. Guideline adherence varied across different professional groups. Significant improvements with adherence by dietitians were noted over time.  Minimal change over time in the overall adherence score from previous survey. Compliance with individual elements of the guideline recommendations comprising the adherence score differed. Improvements in staff practices and attitudes are apparent since the first survey.  Further improvements in guideline awareness and guideline elements are still required to improve the delivery of best practice antenatal GWG care. | Single centre study. Small sample. Survey assessed intention - not behaviour. |
|  | Pauws van Zanten et al. 2017  Netherlands | Quantitative Retrospective observational study. | To study oxygen saturation (SpO2) targeting before and after training and guideline implementation of manual oxygen titration. | Implementation of manual oxygen titration guideline. | Neonatal intensive care unit (NICU). Two cohorts of preterm infants <30 weeks of gestation. Nurses and medical staff. | Enablers Focused training before implementation. Personal engagement of staff during training influenced behaviour change. Open discussion of guideline with caregivers during training sessions.  Factors related to Organization (i.e., support from physicians).  Nurses (i.e., awareness of and attitudes to guidelines)  Anticipated consequences (i.e., benefit to the patients and nurses work).  Patient group (i.e., topic of the guideline) were identified as important factors for adopting a guideline. | Training and guideline implementation approach for quality improvement. To increase awareness in SpO2 targeting and oxygen titration, all nurses were trained in a months period. Training session lasted 30 to 45 minutes. Medical staff were trained during a grand round session.  Education and follow-up with staff by trainers. Medical staff required to monitor/check oxygen saturation distribution routinely during daily rounds. Guideline reviewed with nurses for feedback to make it more practicable to use. | Training and guideline implementation in manual oxygen titration improved SpO2 targeting in preterm infants with more time spent within the target range and less frequent hyperoxaemia.  The durations of hypoxaemia and hyperoxaemia during ABCs were shorter. Extra training and implementing a guideline in oxygen titration improved the compliance of caregivers in NICU in oxygen targeting and a more prompt handling of ABCs (apnoea, bradycardia, cynosis). | Different contextual variables may have influenced the effect of training and results.  Retrospective nature of study. Limited to before and after audit. |
|  | Zahroh et al. 2022  Australia and Switzerland | Systematic review | To assess factors (barriers and facilitators) affecting the appropriate use of antenatal corticosteroids (ACS), tocolytics, magnesium sulphate, and antibiotics to improve preterm birth management. | Use of antenatal corticosteroids (ACS), tocolytics, magnesium sulphate, and antibiotics to improve preterm birth management |  | Identified 8 overarching themes: 1) inaccurate assessment of gestational age;  (2) inconsistent practice guidelines;  (3) variable knowledge about the interventions;  (4) providers perceived risks and benefits;  (5) barriers in administration of interventions;  (6) appropriate settings for administration;  (7) strategies to improve appropriate use; and  (8) womens perspectives and experiences.  Barriers Challenges around accurately assessing gestational age,  inconsistent clinical guidelines and protocols,  healthcare providers variable knowledge of intervention benefits and harms,  and system-level challenges around stock-outs of medicine, limited human resources, and substandard labour and newborn care.  Womens' and partners' lack of knowledge and understanding about preterm birth, its management, risks, side effects, and impact of intervention on baby- contributing to hesitancy.  Enablers- Strategies to improve use Implementing reminder systems and educational materials. Developing reporting indicators and audit and feedback cycles. Implementing education and training for health providers. Appointing change champions. Multidisciplinary teamwork to improve quality of care. Trust and relationship between woman and healthcare providers, support, autonomy and empowerment. | Most included studies were from high-income countries (37/46 studies), which may affect the transferability of these findings to low- or middle-income settings. | See table 3- summary of findings Study findings can inform clinical preterm birth guidelines and implementation to ensure that barriers are addressed, and enablers are reinforced to ensure these interventions are widely available and appropriately used globally.  8 Critical factors affecting implementation of 4 interventions to improve preterm birth management globally. |  |

Data extraction process: Single person data extraction: KR. Data verification: CE (10% of papers), KP verification of extracted data table. Data extracted completed between June 2023 and July 2023 following full-text screening.
