# Supplementary material for: Barriers and enablers for implementation of clinical practice guidelines in maternity and neonatal settings: A rapid review
Source: PLoS One. 2024 Dec 16;19(12):e0315588. doi: 10.1371/journal.pone.0315588 (PMC11649122; doi:10.1371/journal.pone.0315588)
Supplement: S3 File — (DOCX) [file pone.0315588.s003.docx]

**S3 Appendix Expanded overview of study characteristics**

| **Author, year**  **Study origin/ country** | **Study type, methods** | **Purpose/aim** | **Intervention (guideline, policy, recommendation)** | **Setting and participants** | **Quality appraisal percentage of criteria met (QuADS Tool Harrison et al. 2021)** |
| --- | --- | --- | --- | --- | --- |
| Akuma 2012  United Kingdom (UK) | Quantitative Descriptive survey | To examine neonatal analgesia knowledge and guideline application in routine practice | Guideline by Association of Paediatric Anaesthetists of Great Britain and Northern Ireland 2008 | Doctors and nurses.  Neonatal intensive care units (NICU) (n=7) in one area of UK. | 82% |
| Albouy-Llaty et al. 2012  France | Quantitative Retrospective | To evaluate guideline adherence | Perinatal Group B streptococcus (GBS) screening guidelines | Hospitals across a French region.  Woman (n=5997) and neonates (n=84) data.  Midwives and obstetricians. | 85% |
| Alja'freh and Abu-Shaikha 2021  Jordan | Quantitative Cross-sectional survey | To assess adherence to guidelines | Clinical practice guidelines (CPG) of Hypertensive Disorders (HDP) of Pregnancy | Four public, private and military hospitals.  Healthcare providers in maternal health services (n=270). Nurses, midwives and obstetricians. | 90% |
| Alsweiler et al. 2019  New Zealand | Quantitative Retrospective cohort study | To assess if routine calculation of birthweight centiles improved guideline adherence and if adherence correlated with identification of at-risk infants. | Neonatal hypoglycaemia guideline | Tertiary hospital. Maternal and neonatal data.  Infants (n=400). | 82% |
| Breakell 2018  United Kingdom | Quantitative  Before and after audit, Quality improvement project | To investigate guideline implementation accompanied by an educational programme. | National Institute of Health and Care Excellence (NICE) bronchiolitis guideline | District General Hospital (n=1) Clinical Staff.  Patients (50 pre- and 51post-intervention). | 97% |
| Brower et al. 2019  United States | Quantitative Retrospective baseline data evaluation of pre-and post-guideline intervention | To assess the percentage of increase in patients who are tested and treated in accordance with local guideline recommendations. | Local guideline recommendation for treatment of Herpes Simplex Virus (HSV) and use of acyclovir. | Children’s hospital (>650 beds) and satellite location (>40 beds).  0 to 60 days of age, six-month (Jan to June 2014) patient data (n=160) for pre-guideline, and 1120 post-implementation. | 95% |
| Brozanski et al. 2020  United States | Quantitative  Quality improvement project | To describe development and implementation of a standardised process to maintain perioperative euthermia. | Perioperative euthermia clinical practice recommendations | Children's hospital NICUs (n=19) from a Children's Hospital Neonatal Consortium. Preoperative paediatric and neonatal healthcare professionals (physicians, surgeons, anaesthesiologists, nurses). | 92% |
| David et al. 2021  Zimbabwe | Mixed methods study Retrospective medical record data and clinician interviews | To identify gaps requiring service and care delivery improvement for pregnant women visiting a central hospital. | Prenatal care pathways for pregnant women. | Hospital  Maternity case records (n=100) and Interviews (n-20) with maternity care clinicians (nurses, midwives, gynaecologists, obstetricians). | 85% |
| da Silva Carvalho et al. 2021  Brazil | Mixed methods study  Exploratory case study and content analysis | To synthesise stakeholder contributions about guideline implementation from a public consultation. | National Clinical Guidelines for Care in Normal Birth. | Public health system. | 85% |
| de Oliveira Carvalho 2013  Brazil | Qualitative  Interviews | To assess role of nursing team in clinical management of mothers breastfeeding in a maternity setting. | Maternal breastfeeding clinical management of lactation and the  orientation/techniques to prevent early difficulties during  breastfeeding. | Childrens Hospital. Nurses (n=1) and nurse technicians (n=7) involved in supporting breastfeeding. | 77% |
| Doherty et al. 2020  Australia | Quantitative Randomised stepped-wedge controlled trial and self-reported online/telephone interview questionnaire | To assess effectiveness of a practice change intervention towards provision of antenatal care in public maternity services. | Model of care addressing alcohol consumption based on systematic review of evidence, international and Australian clinical guidelines. | Public maternity services and antenatal care services across a health district (urban and regional/rural).  Pregnant woman (n=5694). | 97% |
| Eldh 2016  Sweden | Qualitative (nonparticipant observation and interviews) | To evaluate effectiveness of computer reminders for evidence-based management of peripheral venous catheters (PVC). | Guideline for PVC management in paediatric care. | Paediatric university hospital (approximately 245 beds), 16 units (including emergency, medical, surgical, orthopaedic, NICU), 940 registered nurses. | 95% |
| Gkentzi et al. 2017  United Kingdom | Systematic review | To summarise evidence about antenatal vaccination against pertussis. | Recommendations for national immunisation program- antenatal vaccination against pertussis. | Literature in English from January 2011 to May 2016  searched in four databases. | 90% |
| Gu et al. 2020  China | Quantitative, controlled before-and-after-implementation study | To evaluate guideline implementation and impact on nurses’ behaviour and effect on infants outcomes. | Guideline of Enteral Nutrition for Infants with Congenital Heart  Disease. | Cardiac centre at a childrens hospital.  Infants (n=142) with congenital heart disease and nurses (n=100). | 95% |
| Haskell et al. 2021  New Zealand and Australia | Mixed methods study  Quantitative measurements and qualitative via an online questionnaire | To evaluate effectiveness and uptake of evidence-based bronchiolitis management interventions. | Targeted theory-informed interventions to improve bronchiolitis management in acute paediatric setting. | 26 hospitals in Australia and New Zealand. Emergency and paediatric inpatient units. Intervention (n=13) and control (n=13). Clinicians and clinical leads (nursing and medical). | 100% |
| Kebaya et al. 2018  Kenya | Quantitative. Retrospective baseline audit of medical records and follow-up questionnaire | To evaluate healthcare workers’ compliance with evidence-based criteria for newborn resuscitation. | Evidence-based criteria regarding newborn resuscitation in maternity units. | District hospital maternity units  Healthcare providers in labour ward, maternity theater, postnatal and newborn units. | 100% |
| Langley et al. 2015  Canada | Quantitative cross-sectional survey | To investigate infection prevention and control practices related to Methicillin-Resistant Staphylococcus aureus (MRSA), additional precautions and other practices in paediatric settings. | Canadian Nosocomial Infection Surveillance Program for MRSA transmission in paediatric health care facilities. | 50 hospitals. Infection control professionals working in settings providing care to children in Canada. | 87% |
| Laubscher et al. 2013  Switzerland | Quantitative  questionnaire. | To assess acceptance of guidelines to prevent vitamin K deficiency bleeding by paediatricians. | Swiss guidelines to prevent vitamin K deficiency bleeding. | Swiss Society of Paediatrics (n=629). | 77% |
| Luitjes et al. 2018  Netherlands | Quantitative  Cluster randomised control trial (cRCT) | To evaluate the effectiveness of guidelines on the management of hypertension in pregnancy compared to a common strategy of professional audit and feedback. | Obstetric guidelines on the management of hypertension in pregnancy. | Hospitals providing obstetric care (n=16). Health professionals - obstetricians and gynaecologists, residents and clinical midwives. Patients- pregnant women with hypertensive disorder. | 97% |
| Lyngstad et al. 2021  Norway | Quantitative  Quality improvement project | To assess impact of implemented guidelines for pain assessment and management and increased parental involvement. | Guidelines for pain assessment and management and increased parental involvement in single-family room NICU. | NICU, interprofessional clinical staff, and parents. | 97% |
| Mohan et al. 2023  United States | Quantitative Retrospective | To investigate use of intravenous immunoglobulin (IVIG) in infants with haemolytic disease and guideline compliance. | American Academy of Paediatrics (AAP) guideline recommendations for IVIG in infants with haemolytic disease. | Hospital, NICU (82 beds), infants. | 87% |
| Moore et al. 2020  Canada | Qualitative interview and content analysis | To identify facilitators and barriers to implementation of a shared decision-making guideline. | Guideline supporting shared decision making for extreme preterm birth. | Children's' Hospital. Healthcare Care Providers (16 physicians, nine nurses). | 92% |
| Muhumuza et al. 2015  Uganda | Quantitative  Pre-post (audit) | To investigate hand hygiene compliance among health care workers in the paediatric special care unit. | Hand hygiene to reduce transmission of health care worker-associated pathogens. | Paediatric special care unit of a national referral hospital. Health care workers. | 95% |
| Muirhead and Kynoch 2019  Australia | Quantitative Pre/post audit design | To assess improvement in pain assessment and management in neonates receiving an opioid infusion and incidence of iatrogenic withdrawal syndrome. | Evidence-based clinical guideline for the management of neonatal pain published by the Australian New Zealand Neonatal Network. | Large tertiary referral hospital- two NCCUs (79 beds). | 100% |
| Nair et al. 2014  United Kingdom | Systematic review | To synthesis global information about facilitators and barriers to improving quality of care for pregnant women, newborns and children. | Quality of care | Health systems of all countries. | 92% |
| Nkamba et al. 2017  Zambia and Democratic Republic of Congo (DRC) | Qualitative, group interviews and focus groups | To identify barriers and facilitators to the implementation of antenatal screening and treatment during pregnancy. | Antenatal screening and treatment during pregnancy. | Primary care clinics (n=11). Clinic administrators, health care workers (midwives, nurses, physicians, lab technicians, counsellors and nutritionists), and pregnant woman (n=112). | 92% |
| O'Loughlin et al. 2021  Lao | Quantitative  Pre- and post-training survey | To evaluate redesigned paediatric care training and paediatric care case management model. | Integrated management of neonatal and childhood illness guidelines- national strategy. | Provincial-and district-level hospital staff (n=56).  Health centre workers (nurses, midwives and healthcare assistants (n=43). | 87% |
| Olsen et al. 2018  United States | Quantitative  Retrospective data analysis  Quality Improvement project | To develop standardised nutritional guidelines to promote increased growth velocity in premature infants. | Nutritional guidelines for premature infants. | Children's hospital -NICU (74-beds).  Interdisciplinary NICU team staff. | 95% |
| Page et al. 2017  Australia | Qualitative, semi structured interviews and content analysis | To identify barriers to early optimal nutrition to inform implementation strategies in translational research. | Nutrition guidelines for infants who weigh <1500 gms (preterm birth). | Tertiary-level hospital NCCU. Medical (Consultants, Fellows and Registrars) and nursing staff (experienced, recent graduates and Clinical Nurse Facilitators) (n=19). | 85% |
| Pangerl et al. 2021  Australia | Systematic review | To synthesis information about compliance with GBS screening protocols in a variety of settings. | GBS Screening Guidelines in Pregnancy. | Studies located in maternity homes, private obstetric practice, and hospital clinical environments. | 87% |
| Pauws et al. 2017  Netherlands | Quantitative Retrospective observational study | To examine oxygen saturation (SpO2) targeting before and after training and guideline implementation. | Implementation of manual oxygen titration guideline. | NICU. Two cohorts of preterm infants <30 weeks of gestation. Nurses and medical staff. | 85% |
| Pricilla et al. 2018  Kenya | Quantitative Retrospective study of programmatic data | To assess adoption of prevention of mother to child transmission of Human immunodeficiency virus (HIV) guidelines and associated outcomes. | World Health Organisation's (WHO) Prevention of mother to child transmission of HIV treatment guidelines. | Four government hospitals.  Mother-infant pairs (n=2604) | 92% |
| Rousseau et al. 2020  France | Mixed methods before and after cross-sectional vignette-based survey study | To explore spontaneous preterm birth prevention practices before and after guideline dissemination and identify personal and organisational adherence factors. | National guidelines for obstetrics. | French obstetricians  Public and private maternity units.  Before n=286, after n=282, and both n=145. | 92% |
| Ryan et al. 2020  United States | Systematic review | To synthesis information about implementation outcomes of obstetric haemorrhage (OH) prevention innovations in low- and middle-income countries (LMIC). | PPH clinical guidelines, policy and management of OH prevention. | Obstetrics, LMICs | 100 |
| Sharma et al. 2021  Norway | Qualitative  Focus groups with purposive sample | To examine experiences and perceptions among Nordic and South Asian women with previous gestational diabetes mellitus (GDM). | Lifestyle-changes guidelines essential for preventing diabetes post-GDM. | Three hospital outpatient clinics. Woman 1-3 years after a pregnancy with GDM (n=23) | 97% |
| Silva et al. 2013  Brazil | Quantitative Randomised controlled trial  Before and after intervention | To assess effectiveness of academic detailing of obstetricians versus no intervention on screening of pregnant women for GBS | Guidelines for GBS prenatal screening. | Medical cooperative. Prenatal outpatient care-community. Obstetricians (n=241) | 85% |
| Skare et al. 2018  Norway | Quantitative  Pre-post test | To assess neonatal resuscitations skills and team performance after implementation of video-assisted, performance-focused debriefings. | Neonatal resuscitations skills | University hospital delivery wards.  Compared 74 resuscitation events pre-implementation to 45 events post-implementation. Midwives and physicians. | 92% |
| Smith et al. 2017  United Kingdom | Qualitative  Case studies - secondary analysis | To examine country case studies related to implementation of essential components of maternal death surveillance and response (MDSR) and identify improvements for Maternal Death Review (MDR) systems. | MDSR and MDR systems | 10 case studies -countries as part of World Health Organisation (WHO) Global MDSR Implementation survey. 10 countries at different stages of implementation of Maternal Death Review (MDR) systems. | 92% |
| Snelgrove-Clarke et al.  2015  Canada | Quantitative Randomised controlled trial | To assess Action Learning intervention on nurses’ use of a fetal health surveillance (FHS) guideline during labour of women who were low risk on admission for delivery. | FHS Guideline in Clinical Practice. | Regional tertiary care hospital, perinatal centre. Birthing unit. Nurses (n=89). | 92% |
| Stokes et al. 2016  New Zealand | Systematic review | To synthesise evidence on guideline implementation strategies to improve obstetric care practice in LMICs and identify barriers and enablers for successful implementation. | Guidelines to improve obstetric care practice. | LMICs- Sub-Saharan Africa and in hospital obstetric health care facilities. | 92% |
| Sundercombe et al. 2014  Australia and New Zealand. | Quantitative  Cross-sectional survey and guidelines quality appraisal (using checklist) | To investigate how well postnatal ward neonatal hypoglycaemia guidelines facilitate breast feeding and adhere to UNICEF (United Nations Childrens Fund) UK Baby Friendly Initiative recommendations and different recommendations. | Postnatal ward neonatal hypoglycaemia guidelines and UNICEF UK Baby Friendly Initiative recommendations. | Tertiary neonatal centres (n=23) in Australia and New Zealand. | 92% |
| Telfer et al. 2021  United States. | Quantitative  Chart audits and root-cause analysis | To introduce an evidence-based bundle to reduce early labour admissions and increase guideline adherence. | Evidence-based bundle to reduce early labour admissions and labour management guidelines associated with decreased caesarean birth. | Teaching hospital birthing unit team.  Certified nurse-midwife, obstetrician, resident, student, nurse-midwife, pediatric provider, scrub technician, three to four nurses, and an anaesthesia team. | 100% |
| Trevisanuto et al. 2015  Vietnam | Quantitative Survey | To investigate consistency of resuscitation practices and adherence with international neonatal resuscitation guidelines. | International guidelines for neonatal resuscitation. | Public central, provincial and district hospitals (n=160) representing the three levels of public hospital-based maternity services. | 95% |
| Trollope et al. 2018  New Zealand | Quantitative  Questionnaire and  Appraisal of Guidelines Research & Evaluation | To investigate factors related to poor compliance with maternity guidelines. | Maternity clinical practice guidelines developed by National Womens Health. | 10 purposively selected guidelines related to maternal and neonatal care. Clinicians (n=82). | 90% |
| Turan et al. 2012  Kenya | Quantitative  Cluster randomised controlled trial | To examine effects of integrating HIV treatment into Antenatal Care (ANC) clinics at public health facilities. | HIV and ANC Integration in Pregnancy in Kenya | Rural government health facilities (n=12). Pregnant HIV-positive woman. | 90% |
| Warren 2011  Australia | Pre- and post-implementation audit strategy | To investigate nurses understanding and management of infants with intravenous therapy. | Protocol for the prevention and management of extravasation injuries in the neonatal intensive care. | NCCU (79-cots) of a large tertiary level hospital. | 92% |
| Wilkinson et al. 2017  Australia | Quantitative  Cross-sectional prospective online survey | To re-evaluate staff knowledge, attitudes and behaviours around the management of gestational weight gain (GWG) following service changes. | Clinical guidelines regarding weight management in pregnancy- best practice delivery of care to pregnant women regarding GWG. | Tertiary maternity hospital. Antenatal staff (obstetricians, midwives and allied health) (n=69) | 87% |
| Zahroh et al. 2022  Australia and Switzerland | Systematic review | To assess barriers and facilitators affecting the appropriate use of antenatal corticosteroids, tocolytics, magnesium sulphate, and antibiotics to improve preterm birth management. | Use of antenatal corticosteroids, tocolytics, magnesium sulphate, and antibiotics to improve preterm birth management |  | 92% |

Abbreviations: American Academy of Paediatrics AAP; Antenatal Care ANC; Clinical practice guidelines CPG; Foetal health surveillance FHS; Gestational diabetes mellitus GDM; Gestational weight gain GWG; Group B streptococcus GBS; Herpes Simplex Virus HSV; Human immunodeficiency virus HIV; Hypertensive Disorders HDP; Intravenous immunoglobulin IVIG; Low- and middle-income countries LMIC; Methicillin-Resistant Staphylococcus aureus MRSA; National Institute of Health and Care Excellence NICE; Neonatal Critical Care Unit NCCU; Neonatal intensive care unit NICU; peripheral venous catheters PVC; Postpartum haemorrhage PPH; Obstetric haemorrhage OH; Quality assessment with diverse studies QuADS; United Nations Childrens Fund UNICEF; United Kingdom UK; World Health Organisation WHO.
