# Supplementary material for: Barriers and enablers for implementation of clinical practice guidelines in maternity and neonatal settings: A rapid review
Source: PLoS One. 2024 Dec 16;19(12):e0315588. doi: 10.1371/journal.pone.0315588 (PMC11649122; doi:10.1371/journal.pone.0315588)
Supplement: S4 File — (DOCX) [file pone.0315588.s004.docx]

**S4 Appendix Quality appraisal**

| No. | Reviewer/Rater | Study/Article | Type of study/project | 1. Theoretical or conceptual underpinning to the research | 2. Statement of research aim/s | 3. Clear description of research setting and target population | 4. The study design is appropriate to address the stated research aim/s | 5. Appropriate sampling to address the research aim/s | 6. Rationale for choice of data collection tool/s | 7. The format and content of data collection tool is appropriate to address the stated research aim/s | 8. Description of data collection procedure | 9. Recruitment data provided | 10. Justification for analytic method selected | 11. The method of analysis was appropriate to answer the research aim/s | 12. Evidence that the research stakeholders have been considered in research design or conduct. | 13. Strengths and limitations critically discussed | TOTAL | Percent of criteria met | Moderation CE /KR  Verification KP |
| --- | --- | --- | --- | --- | --- | --- | --- | --- | --- | --- | --- | --- | --- | --- | --- | --- | --- | --- | --- |
| 1 | KR | Albouy-Llaty et al. 2012 | Retrospective | 2 | 3 | 3 | 3 | 3 | 3 | 3 | 3 | 3 | 3 | 3 | 0 | 0 | 32 | 82% | moderated 25/08/2023 |
|  | CE | Interrater |  | 3 | 3 | 3 | 3 | 3 | 3 | 3 | 3 | 3 | 3 | 3 | 0 | 0 | 33 | 85% |  |
| 2 | KR | Alja'freh"and Abu-Shaikha 2021 | Cross sectional study | 2 | 2 | 3 | 2 | 3 | 2 | 2 | 3 | 3 | 3 | 3 | 2 | 2 | 32 | 82% | moderated 25/08/2023 |
|  | CE | Interrater |  | 3 | 3 | 3 | 3 | 3 | 2 | 3 | 3 | 3 | 3 | 3 | 2 | 2 | 36 | 92% |  |
| 3 | KR | Alsweiler et al. 2019 | Retrospective | 2 | 3 | 3 | 3 | 3 | 3 | 3 | 3 | 3 | 3 | 2 | 0 | 0 | 31 | 79% | moderated 25/08/2023 |
|  | CE | Interrater |  |  |  |  |  |  |  |  |  |  |  |  |  |  |  |  |  |
| 4 | KR | Akuma 2012 | Descriptive survey | 3 | 3 | 3 | 2 | 3 | 3 | 2 | 3 | 3 | 3 | 2 | 0 | 2 | 32 | 82% | moderated 25/08/2023 |
|  | CE | Interrater |  | 3 | 3 | 3 | 3 | 3 | 3 | 3 | 3 | 3 | 3 | 3 | 0 | 3 | 36 | 92% |  |
| 5 | KR | Breakell 2018 | Before and after | 3 | 3 | 3 | 3 | 3 | 3 | 2 | 3 | 3 | 3 | 2 | 0 | 3 | 34 | 87% | moderated 25/08/2023 |
|  | CE | Interrater |  | 3 | 3 | 3 | 3 | 3 | 3 | 3 | 3 | 3 | 3 | 3 | 3 | 3 | 39 | 100% |  |
| 6 | KR | Brower et al. 2019 | Retrospective | 3 | 3 | 3 | 2 | 3 | 3 | 2 | 3 | 3 | 3 | 3 | 3 | 3 | 37 | 95% | moderated 25/08/2023 |
|  | CE | Interrater |  | 2 | 3 | 3 | 2 | 3 | 3 | 2 | 3 | 3 | 3 | 3 | 3 | 3 | 36 | 92% |  |
| 7 | KR | Brozanski et al. 2020 | Quantitative data | 2 | 3 | 3 | 3 | 3 | 3 | 2 | 3 | 3 | 3 | 3 | 3 | 3 | 37 | 92% |  |
| 8 | KR | David et al. 2021 | Mixed methods | 3 | 3 | 3 | 3 | 3 | 3 | 3 | 3 | 3 | 3 | 3 | 0 | 0 | 33 | 85% |  |
| 9 | KR | de Silva Carvalho et al. 2021 | Mixed methods | 3 | 3 | 3 | 2 | 2 | 3 | 2 | 3 | 3 | 3 | 3 | 0 | 3 | 33 | 85% |  |
| 10 | KR | deOliveiraCarvalho 2013 | Qualitative | 3 | 3 | 3 | 3 | 2 | 3 | 3 | 2 | 1 | 3 | 3 | 0 | 0 | 29 | 77% |  |
| 11 | KR | Doherty et al. 2020 | Randomised control | 3 | 3 | 3 | 3 | 3 | 3 | 3 | 3 | 3 | 3 | 3 | 2 | 3 | 38 | 97% |  |
| 12 | KR | Eldh 2016 | Qualitative | 3 | 3 | 3 | 3 | 3 | 3 | 3 | 3 | 3 | 3 | 3 | 2 | 2 | 37 | 95% |  |
| 13 | KR | Gkentzi et al. 2017 | Systematic review | 3 | 3 | 3 | 3 | 3 | 3 | 3 | 3 | 3 | 3 | 3 | 2 | 0 | 35 | 90% |  |
| 14 | KR | Gu et al. 2020 | Before and after-controlled | 3 | 3 | 3 | 3 | 2 | 3 | 3 | 3 | 3 | 3 | 3 | 2 | 3 | 37 | 95% |  |
| 15 | KR | Haskell et al. 2021 | Mixed methods- cRCT | 3 | 3 | 3 | 3 | 3 | 3 | 3 | 3 | 3 | 3 | 3 | 3 | 3 | 39 | 100% |  |
| 16 | KR | Kebaya et al. 2018 | Retrospective | 3 | 3 | 3 | 3 | 3 | 3 | 3 | 3 | 3 | 3 | 3 | 3 | 3 | 39 | 100% |  |
| 17 | KR | Keely, 2012 | Literature review | 1 | 1 | 0 | 1 | 1 | 1 | 1 | 0 | 0 | 0 | 1 | 0 | 0 | 7 |  | Consensus Exclude this paper |
| 18 | KR | Langley et al. 2015 | Cross sectional survey | 3 | 3 | 3 | 3 | 3 | 3 | 3 | 3 | 3 | 3 | 3 | 1 | 0 | 34 | 87% |  |
| 19 | KR | Laubscher et al. 2013 | Quantitative, questionnaire. | 1 | 2 | 3 | 3 | 3 | 3 | 3 | 2 | 1 | 3 | 3 | 0 | 2 | 29 | 77% |  |
| 20 | KR | Luitjes et al. 2018 | Cluster randomised control trial (cRCT) | 3 | 3 | 3 | 3 | 3 | 3 | 3 | 3 | 3 | 3 | 3 | 2 | 3 | 38 | 97% |  |
| 21 | KR | Lyngstad et al. 2021 | Retrospective | 3 | 3 | 3 | 3 | 3 | 3 | 3 | 3 | 3 | 3 | 3 | 2 | 3 | 38 | 97% |  |
| 22 | KR | Mohan et al. 2023 | Retrospective study. | 3 | 2 | 3 | 3 | 3 | 3 | 3 | 3 | 3 | 3 | 3 | 2 | 0 | 34 | 87% |  |
| 23 | KR | Moore et al. 2020 | Qualitative | 3 | 1 | 3 | 3 | 3 | 3 | 3 | 3 | 3 | 3 | 3 | 2 | 3 | 36 | 92% |  |
| 24 | KR | Muhumuza et al. 2015 | pre-post (audit) | 3 | 3 | 3 | 3 | 3 | 3 | 3 | 3 | 2 | 3 | 3 | 2 | 3 | 37 | 95% |  |
| 25 | KR | Muirhead and Kynoch 2019 | pre/post audit design | 3 | 3 | 3 | 3 | 3 | 3 | 3 | 3 | 3 | 3 | 3 | 3 | 3 | 39 | 100% |  |
| 26 | KR | Nair et al. 2014 | Systematic literature review. | 3 | 3 | 3 | 3 | 3 | 3 | 3 | 3 | 3 | 3 | 3 | 0 | 3 | 36 | 92% |  |
| 27 | KR | Nkamba et al. 2017 | Qualitative | 3 | 3 | 3 | 3 | 3 | 3 | 3 | 3 | 3 | 3 | 3 | 0 | 3 | 36 | 91% |  |
| 28 | KR | O'Loughlin et al. 2021 | Pre- and post-training survey. | 3 | 3 | 3 | 3 | 3 | 3 | 3 | 3 | 3 | 3 | 3 | 2 | 3 | 38 | 97% |  |
| 29 | KR | Olsen et al. 2018 | Retrospective data | 3 | 2 | 3 | 3 | 3 | 3 | 3 | 3 | 3 | 3 | 3 | 2 | 3 | 37 | 95% |  |
| 30 | KR | Page et al. 2017 | Qualitative | 3 | 3 | 3 | 3 | 3 | 3 | 3 | 3 | 3 | 3 | 3 | 0 | 0 | 33 | 85% |  |
| 31 | KR | Pangerl et al. 2021 | Systematic literature review | 3 | 3 | 3 | 3 | 3 | 3 | 3 | 3 | 3 | 3 | 3 | 0 | 1 | 34 | 87% |  |
| 32 | KR | Pricilla et al. 2018 | Retrospective | 3 | 3 | 3 | 3 | 3 | 3 | 3 | 3 | 3 | 3 | 3 | 0 | 3 | 36 | 92% |  |
| 33 | KR | Rousseau et al. 2020 | Mixed methods | 3 | 3 | 3 | 3 | 3 | 3 | 3 | 3 | 3 | 3 | 3 | 0 | 3 | 36 | 92% |  |
| 34 | KR | Ryan et al. 2020 | Systematic review | 3 | 3 | 3 | 3 | 3 | 3 | 3 | 3 | 3 | 3 | 3 | 3 | 3 | 39 | 100% |  |
| 35 | KR | Sharma et al. 2021 | Qualitative | 3 | 3 | 3 | 3 | 3 | 3 | 3 | 3 | 3 | 3 | 3 | 2 | 3 | 38 | 97% |  |
| 36 | KR | Silva et al. 2013 | Randomised controlled trial (RCT) | 3 | 3 | 3 | 3 | 3 | 3 | 3 | 3 | 3 | 3 | 3 | 0 | 0 | 33 | 85% |  |
| 37 | KR | Skare et al. 2018 | Quantitative, Pre-post test | 3 | 3 | 3 | 3 | 3 | 3 | 3 | 3 | 3 | 3 | 3 | 0 | 3 | 36 | 92% |  |
| 38 | KR | Smith et al. 2017 | Case studies - secondary analysis | 3 | 3 | 3 | 3 | 3 | 3 | 3 | 3 | 3 | 3 | 3 | 0 | 3 | 36 | 92% |  |
| 39 | KR | Snelgrove-Clarke et al. 2015 | Quantitative, randomized controlled trial. | 3 | 3 | 3 | 3 | 3 | 3 | 3 | 3 | 3 | 3 | 3 | 0 | 3 | 36 | 92% |  |
| 40 | KR | Stokes et al. 2016 | Systematic review | 3 | 3 | 3 | 3 | 3 | 3 | 3 | 3 | 3 | 3 | 3 | 0 | 3 | 36 | 92% |  |
| 41 | KR | Sundercombe et al. 2014 | Cross-sectional survey | 3 | 3 | 3 | 3 | 3 | 3 | 3 | 3 | 3 | 3 | 3 | 0 | 3 | 36 | 92% |  |
| 42 | KR | Telfer et al. 2021 | Chart audits and root-cause analysis. | 3 | 3 | 3 | 3 | 3 | 3 | 3 | 3 | 3 | 3 | 3 | 3 | 3 | 39 | 100% |  |
| 43 | KR | Trevisanuto et al. 2015 | Quantitative Survey | 3 | 3 | 3 | 3 | 3 | 3 | 3 | 3 | 2 | 3 | 3 | 2 | 3 | 37 | 95% |  |
| 44 | KR | Trollope et al. 2018 | Quantitative questionnaire. | 3 | 3 | 3 | 3 | 3 | 3 | 3 | 3 | 2 | 3 | 3 | 0 | 3 | 35 | 90% |  |
| 45 | KR | Turan et al. 2012 | cluster randomised controlled trial. | 3 | 3 | 3 | 3 | 3 | 3 | 3 | 3 | 2 | 3 | 3 | 0 | 3 | 35 | 90% |  |
| 46 | KR | Warren 2011 | Pre- and post- audit | 3 | 3 | 3 | 3 | 3 | 3 | 3 | 3 | 2 | 3 | 3 | 3 | 1 | 36 | 92% |  |
| 47 | KR | Wlkinson et al. 2017 | Cross-sectionals survey. | 3 | 1 | 3 | 3 | 3 | 3 | 3 | 3 | 3 | 3 | 3 | 0 | 3 | 34 | 87% |  |
| 48 | KR | Pauws et al. 2017 | Retrospective | 2 | 3 | 3 | 2 | 3 | 3 | 2 | 3 | 3 | 3 | 3 | 0 | 3 | 33 | 85% |  |
| 49 | KR | Zahroh et al. 2022 | Systematic review | 3 | 3 | 3 | 3 | 3 | 3 | 3 | 3 | 3 | 3 | 3 | 0 | 3 | 36 | 92% |  |

Notes- Points assigned -scoring -QuADS criteria 0 1 2 3; Maximum 39 (100%).
