# Supplementary material for: Barriers and enablers for implementation of clinical practice guidelines in maternity and neonatal settings: A rapid review
Source: PLoS One. 2024 Dec 16;19(12):e0315588. doi: 10.1371/journal.pone.0315588 (PMC11649122; doi:10.1371/journal.pone.0315588)
Supplement: S5 File — (DOCX) [file pone.0315588.s005.docx]

**S5 Appendix Collated summary of key themes related to barriers and enablers for clinical practice guideline implementation**

| Construct - level of impact or application | Barrier | Enabler |
| --- | --- | --- |
| Health care system and systemic factors | Lack of resources (low resource settings)  Models of care  Poor communication and coordination within health care system  Macro-micro level factors  Overburdened health system  Conflicting priorities and lack of policies | Healthcare system structure, services, and delivery  Healthcare priorities, public health resources, and investment  Practice, regulation, standards, incentivisation |
| Patient and population (Women and community) | Costs and resource limitations  Social and cultural influences  Lack of health literacy  Patient factors (beliefs, preference, practices)  Lack of stakeholder involvement | Patient attributes, experience and engagement  Resources and support for patients |
| Guidelines and standards | Multiple or different guidelines in use  Guidelines availability and access  Complexity and applicability of guidelines  Variability in guideline development and quality  Lack of clear benchmarks or standards for practice  Contextual implementation challenges | Guideline quality  Design, accessibility and usability of guidelines  Guidelines development and responsibility |
| Organisational capacity (healthcare organisation, service, or facility) | Resource limitations  Practice variations in organisations  Work design  Environmental and contextual factors  Organisation’s capabilities  Lack of team communication and collaboration  Inadequate dissemination and communication of guidelines  Data collection, monitoring and data management systems in use  Guideline implementation process challenges | Quality improvement initiatives, periodic audit and monitoring  Organisational support, extent of stakeholder engagement in guideline development and implementation process  Dissemination of information, training for clinicians, and promoting awareness of guideline recommendations  Effective work design and decision support mechanisms for clinicians  Health data management systems and processes |
| Health professional practice (clinicians) | Lack of guidelines awareness, and knowledge and skills deficit  Professional motivation and low engagement  Resistance to change  Health workers’ knowledge, attributes, beliefs, attitudes and capability  Lack of interdisciplinary communication and collaboration  Time constraints and workload  Quality of reporting and documentation  Lack of education and training about guidelines and updates | Practice autonomy  Interdisciplinary collaboration and communication  Established practice standards and expectations  Education, training, and feedback  Role, professional identify and involvement in planning and service delivery  Health professionals’ awareness, beliefs, values, attitudes and positive behaviour change |
