# Supplementary material for: Barriers and enablers for implementation of clinical practice guidelines in maternity and neonatal settings: A rapid review
Source: PLoS One. 2024 Dec 16;19(12):e0315588. doi: 10.1371/journal.pone.0315588 (PMC11649122; doi:10.1371/journal.pone.0315588)
Supplement: Attachment — Submitted filename: Manuscript review PONE-D-24-16268_mb 2024 10 15.pdf [file pone.0315588.s006.pdf]

PlosOne PONE-D-24-16268 Review of manuscript

Thank you for the opportunity to review the manuscript titled, *Barriers and enablers for implementation of clinical practice guidelines in maternity and neonatal settings: A rapid review.* This is a well-written paper and highlights the inconsistencies of guideline implementation for maternal and neonatal care. There are a few suggestions for the authors to consider before publication is considered, as follows:

Line 138 Table 1. The authors have used neonatal* as a search term in PubMed. Is there a reason the term, *neonat**, in preference to *neonatal** was used? The term, *neonat**, would have yielded more results.

In view of the study aim, I am interested to know why the authors haven't included *intrapartum* as a search term.

Line 153. It would be beneficial to include Figure 1 in the manuscript for reviewers.

Line 211-212 The International Confederation of Midwives uses the language, *woman* and *women*. Why has the term, *person-centred care* been used when discussing maternity models of care as opposed to *women/woman-centred care?*

Line 199 & 384 (Table 4) Barriers and enablers to guideline implementation for maternity & neonatal settings. Have the authors considered the approach by Squires et al. (2023) to frame the discussion? https://doi.org/10.1186/s12961-023-01028-z

Line 227 The authors identified the lack of women/support person/parents of neonates' *involvement* in guideline development as a barrier. The point is then repeated in Table 4 (page 21) but the language modified. My query is whether healthcare recipients are *involved* in guideline development, or do they *influence* their development? Should the language be modified line 227?

Line 240 - 242. Did continuity of care models emerge as influential in *establishing trust* and *rapport*, and with *decision-making*?

Page 22. *Practice variations in organisations*: was this finding due to differences within the health professional workforce influencing philosophies of care and scope of practice? For example, nurses and paediatricians predominantly provide care in neonatal units as opposed to midwives and obstetricians in maternity settings. If so, may need further clarification.

Page 23. Resistance to change: how does *loss of autonomy* and *resistance to change* fit within the one attribute? Do health professionals fear a loss of autonomy due to change?

Page 24. Resources and support for patients: references are listed which is inconsistent with the rest of the document's formatting.

The adoption (and lack of) of WHO Baby Friendly Hospital Initiative guidelines in some clinical settings have not been included in the discussion. Why not?

Suggest further editing to improve the readability of this manuscript.
