# Supplementary material for: Barriers and enablers for implementation of clinical practice guidelines in maternity and neonatal settings: A rapid review
Source: PLoS One. 2024 Dec 16;19(12):e0315588. doi: 10.1371/journal.pone.0315588 (PMC11649122; doi:10.1371/journal.pone.0315588)
Supplement: Attachment — Submitted filename: ResponsetoReviewers-27-Nov-2024.docx [file pone.0315588.s007.docx]

PlosOne PONE-D-24-16268

The authors thank the reviewers for their valuable comments and suggestions to improve the manuscript. These have all been addressed, as detailed in the Table below.

**Author responses:**

| Line 138 Table 1. The authors have used neonatal* as a search term in PubMed. Is there a reason the term, *neonat**, in preference to *neonatal** was used? The term, *neonat**, would have yielded more results. | *Thank you for highlighting “neonate*”*  *The search terms used followed suggested MeSH© (Medical Subject Headings) terms and preliminary PubMed filter suggestions after a preliminary broad search for all the terms identified in the aim. For future searches we will incorporate the suggested term which hadn’t been considered at the time.* |
| --- | --- |
| In view of the study aim, I am interested to know why the authors haven’t included *intrapartum* as a search term. | *Thank you for highlighting “intrapartum”. The search terms used followed suggested MeSH© (Medical Subject Headings) terms and preliminary PubMed filter suggestions after a preliminary broad search for all the terms identified in the aim. For future searches we will incorporate broader search terms.* |
| Line 153. It would be beneficial to include Figure 1 in the manuscript for reviewers. | *Figure 1 was previously submitted -on page 46 after references.*  *Figure 1 is now inserted under Study selection and screening section in the manuscript.* |
| Line 211-212 The International Confederation of Midwives uses the language, *woman* and *women*. Why has the term, *person-centred care* been used when discussing maternity models of care as opposed to *women/woman*-*centred care?* | *We changed “person” to “woman” where this related directly to maternity care.*  *(Table 4: Models of care)*  *The word “person” was retained on one occasion to encompass the woman, parent and/or support persons for lines 392-393: “Other enablers for practice change and guideline adherence were ensuring clinicians’ autonomy and ability to deviate from guidelines where person-centred care warranted change …”* |
| Line 199 & 384 (Table 4) Barriers and enablers to guideline implementation for maternity & neonatal settings. Have the authors considered the approach by Squires et al. (2023) to frame the discussion? https://doi.org/10.1186/s12961-023-01028-z | *Authors thank the reviewer for suggesting Squires et al’s (2023) Implementation context framework. The current rapid review followed Ritchie and Spencer’s thematic framework as per the protocol to guide discussion based on emergent main themes. For future work we may incorporate Squire et al’s approach to frame the discussion.* |
| Line 227 The authors identified the lack of women/support person/parents of neonates’ *involvement* in guideline development as a barrier. The point is then repeated in Table 4 (page 21) but the language modified. My query is whether healthcare recipients are *involved* in guideline development*,* or do they *influence* their development? Should the language be modified line 227? | *True shared decision-making allows for both involvement and influence of all related parties. Although not an element specifically sought out in this rapid review, the senior author (CE) has experience with a consumer representative’s inclusion in the membership of the hospital guideline development group.*  *At the level of WHO, a Guideline Development Group (GDG) includes a patient/consumer representative (for example, p4 of the “WHO recommendations on antenatal care for a positive pregnancy experience” 2016 notes that “The GDG was a diverse group of individuals with expertise in research, guideline development methods, and clinical policy and programmes relating to interventions for ANC and service delivery, also including a patient/consumer representative.” CE is also a member of the GDG for a number of WHO recommendations, which all include one or more consumers. CE recalls consumers having an equal voice in the deliberations, without being able to specifically refer to their “influence” or “involvement”.*  *As we are unable to formally distinguish between these terms within the scope of this review, we have edited lines 241-242 to now read: “Barriers included lack of influence or direct involvement in guideline development and shared decision-making…”* |
| Line 240 - 242. Did continuity of care models emerge as influential in *establishing trust* and *rapport*, and with *decision-making*? | *Surprisingly, this didn’t come up in the publications. Intuitively, it makes sense to examine this. We have now added comment about this at lines 447-449 of the Discussion.* |
| Page 22*. Practice variations in organisations*: was this finding due to differences within the health professional workforce influencing philosophies of care and scope of practice? For example, nurses and paediatricians predominantly provide care in neonatal units as opposed to midwives and obstetricians in maternity settings. If so, may need further clarification. | *Thank you for considering this interesting perspective. Philosophical differences between the relevant professionals didn’t arise in researchers’ reports on barriers and enablers. Whilst we consider this is beyond the scope of the present review, it could be an area for further exploration in future studies of barriers and enablers. We have made comment to this effect at lines 460-461.* |
| Page 23. Resistance to change: how does *loss of autonomy* and *resistance to change* fit within the one attribute? Do health professionals fear a loss of autonomy due to change? | *These two areas of focus had been listed in the one sentence, with all the relevant references collated to be cited at the end of the sentence.*  *The sentence structure has been edited (lines 365-366) to separate the citations for the two separate areas of focus.* |
| Page 24. Resources and support for patients: references are listed which is inconsistent with the rest of the document’s formatting. | *References reformatted correctly.* |
| The adoption (and lack of) of WHO Baby Friendly Hospital Initiative guidelines in some clinical settings have not been included in the discussion. Why not? | *This is an interesting observation. We had not specified aspects of care in the search strategy, such as breastfeeding, infant feeding, analgesia, oral intake in labour, and so on. Specific aspects of care were covered by research that related to our broad aim of “To identify barriers and enablers for the implementation and adoption of clinical practice guidelines in maternal and neonatal settings”. To that end, breastfeeding was included in a small number of research articles. A review of every aspect of maternity or neonatal care is beyond the scope here. Others may take the learnings from our rapid review and apply them in the implementation of guidelines for specific aspects of care, including the importance of guidelines that support the BFHI.* |
| Suggest further editing to improve the readability of this manuscript. | *We have made edits to improve the readability of the manuscript.* |

**Additional requirements**

**Author responses:**

| 1. Please ensure that your manuscript meets PLOS ONE's style requirements, including those for file naming. The PLOS ONE style templates can be found at  [https://journals.plos.org/plosone/s/file?id=wjVg/PLOSOne_formatting_sample_main_body.pdf](https://url.au.m.mimecastprotect.com/s/htwBCzvO0KU2D8JziKsvF9YwBd?domain=journals.plos.org) and  [https://journals.plos.org/plosone/s/file?id=ba62/PLOSOne_formatting_sample_title_authors_affiliations.pdf](https://url.au.m.mimecastprotect.com/s/WKR8CANZyPfW0r75IQt3FGE33_?domain=journals.plos.org) | *Thank you for the guidance. The Manuscript style has been updated as per guide* |
| --- | --- |
| 2. Please identify your study as "systematic review" in the title of your manuscript. | *Amended title as:*  *Barriers and enablers for implementation of clinical practice guidelines in maternity and neonatal settings: A systematic rapid review*  *Our preference, however, is to leave the word “systematic” out. A Rapid Review is one way of undertaking a structured literature review.*  *Article titles in PLOSOne and other journals include a mix of including the word “systematic” in the titles of scoping and rapid reviews. To be truly “systematic” we feel that the methods would have been even more exhaustive. For example, use of Cochrane Risk of Bias tool, GRADE, etc. We would not wish to imply this level of systematic review. We will comply with the editorial decision on this matter, however.* |
| *3. As required by our policy on Data Availability, please ensure your manuscript or supplementary information includes the following:* | |
| A numbered table of all studies identified in the literature search, including those that were excluded from the analyses. | *All studies identfied for full text screening with reasons for exlusion are provdied in Supplementary File S1 Full text screening summary* |
| For every excluded study, the table should list the reason(s) for exclusion. | *Supplementary File S1 Full text screening summary includes the exclusion criteria key- reasons for exclusion at the end of the table.* |
| If any of the included studies are unpublished, include a link (URL) to the primary source or detailed information about how the content can be accessed. | *Not applicable* |
| A table of all data extracted from the primary research sources for the systematic review and/or meta-analysis. The table must include the following information for each study: | *Supplementary File S2 Data extraction includes all extracted data.* |
| Name of data extractors and date of data extraction | *Supplementary File S2 Data extraction.*  *Included at the end of the table. Due to the volume of studies included data extraction was completed between June 2023 and July 2023. Data were extracted by a single reviewer with second reviewer verification of 10% of the studies as per protocol.* |
| Confirmation that the study was eligible to be included in the review. | *Supplementary File S1 Full text screening*  *As per Inclusion and exclusion criteria.* |
| All data extracted from each study for the reported systematic review and/or meta-analysis that would be needed to replicate your analyses. | *Supplementary File S2 Data extraction includes all extracted data.*  *Supplementart File S3 Expanded overview of study characteristics* |
| If data or supporting information were obtained from another source (e.g. correspondence with the author of the original research article), please provide the source of data and dates on which the data/information were obtained by your research group. | *Not applicable* |
| If applicable for your analysis, a table showing the completed risk of bias and quality/certainty assessments for each study or outcome.  Please ensure this is provided for each domain or parameter assessed. For example, if you used the Cochrane risk-of-bias tool for randomized trials, provide answers to each of the signalling questions for each study. If you used GRADE to assess certainty of evidence, provide judgements about each of the quality of evidence factor. This should be provided for each outcome. | *Risk of bias conducted using QuADs, Harrison et al., 2021*  *Supplementary File S4 Quality appraisal* |
| An explanation of how missing data were handled. | *Not applicable for this study.* |
| This information can be included in the main text, supplementary information, or relevant data repository. Please note that providing these underlying data is a requirement for publication in this journal, and if these data are not provided your manuscript might be rejected. | *Information provided in supplementary files* |
| 4. Thank you for stating the following financial disclosure:  "The review activities were funded by Safer Care Victoria, Australia."    Please state what role the funders took in the study.  If the funders had no role, please state: ""The funders had no role in study design, data collection and analysis, decision to publish, or preparation of the manuscript.""  If this statement is not correct you must amend it as needed.  Please include this amended Role of Funder statement in your cover letter; we will change the online submission form on your behalf. | *The funder had no role in the study: added statement*  *We also recognise the omission of another funding source, so that the revised wording on the Title Page and Cover Letter now reads as:*  *“The review activities were funded by Safer Care Victoria, and La Trobe University Alumni Fund, Australia. The funders had no role in study design, data collection and analysis, decision to publish, or preparation of the manuscript.”* |
| 5. Please include a separate caption for each figure in your manuscript. | *Inserted caption for figure 1* |
| 6. Please include captions for your Supporting Information files at the end of your manuscript, and update any in-text citations to match accordingly. Please see our Supporting Information guidelines for more information: [http://journals.plos.org/plosone/s/supporting-information](https://url.au.m.mimecastprotect.com/s/1GFHCBNZzLf4qPENh1uPF2w5b_?domain=journals.plos.org). | *Inserted captions for supplementary files (SI to S5)* |
| 7. Please review your reference list to ensure that it is complete and correct. If you have cited papers that have been retracted, please include the rationale for doing so in the manuscript text or remove these references and replace them with relevant current references. Any changes to the reference list should be mentioned in the rebuttal letter that accompanies your revised manuscript. If you need to cite a retracted article, indicate the article’s retracted status in the References list and also include a citation and full reference for the retraction notice. | *The references list has been checked and corrected. One reference was incorrectly cited, and another was duplicated. These errors have been corrected.*  *1. An excluded paper was incorrectly cited in the originally submitted paper (ref no. 41). It has now been removed:*  *Keely E. An opportunity not to be missed--how do we improve postpartum screening rates for women with gestational diabetes? Diabetes/Metabolism Research & Reviews. 2012;28(4):312-6.*  *2. The originally submitted references numbered 60 and 70 were the same paper. This has been amended:*  *Kebaya LMN, Kiruja J, Maina M, Kimani S, Kerubo C, McArthur A, et al. Basic newborn resuscitation guidelines for healthcare providers in Maragua District Hospital: a best practice implementation project. JBI Database System Rev Implement Rep. 2018;16(7):1564-81.*  *3. A new reference (74) was added to support the discussion related to continuity of care models:*  *Sandall J, Fernandez Turienzo C, Devane D, Soltani H, Gillespie P, Gates S, et al. Midwife continuity of care models versus other models of care for childbearing women. Cochrane database of systematic reviews. 2024;2024(4):CD004667-CD.* |
